# Diet Formats, Feeding Practices, and Environmental Factors Associated with Overweight, Underweight, and Ideal Body Condition in Household Cats

**DOI:** 10.3390/ani16213406

**Published:** 2026-10-28

**Authors:** LeeAnn M. Perry, Justin Shmalberg, Ryan W. Honaker

**Affiliations:** 1 Research & Development, KatKin (Anikin Ltd.), London EC1R 4RB, UK; 2 Department of Comparative, Diagnostic, and Population Medicine, College of Veterinary Medicine, University of Florida, Gainesville, FL 32610, USA

**Keywords:** feline obesity, overweight, underweight, owner-reported body condition score, companion animal nutrition, cross-sectional study, veterinary epidemiology

## Abstract

This exploratory cross-sectional study analyzed owner-reported survey data from 5653 cats belonging to current and prospective customers of a UK fresh cat food company to identify factors associated with body condition in companion cats and to compare owner classifications made using a structured body condition score (BCS) tool with those made by unguided judgment. Owners classified fewer cats as ideal using the structured BCS tool than by unguided judgment, with lower agreement for underweight than for overweight cats. Multivariable logistic regression models were developed for overweight, underweight, and ideal body condition. A higher proportion of fresh food was associated with greater odds of ideal body condition (OR 1.22, 95% CI: 1.04–1.42), while dry food was associated with overweight (OR 1.83, 95% CI: 1.33–2.51) and wet food with underweight (OR 1.51, 95% CI: 1.06–2.16), though this association was less robust to differing model specifications. Further associations included certain additional calorie sources, feeding practices, treat type, activity, enrichment, household composition, and established demographic factors. These hypothesis-generating findings highlight a gap between structured and unguided owner assessment and identify associations that merit prospective evaluation in a more generally representative population.

## Simple Summary

Cat owners often struggle to tell whether their cat is too heavy, too thin, or a healthy weight, which can delay intervention. In this study, 5653 current and prospective customers of a fresh food company in the United Kingdom completed surveys about their cats’ diet, activity, home environment, and body shape. We assessed which everyday factors are linked to cats being overweight, underweight, or a healthy weight, and whether owners’ classifications differed when using a structured scoring guide rather than unguided judgment. We found that owners identified fewer cats as a healthy weight when using a structured scoring guide than when judging by eye alone. We also found that fresh, mildly cooked food was modestly linked to cats being a healthy weight, while dry food was linked to being overweight and wet food was linked to being underweight, though less consistently. Other everyday factors, including how often cats were fed and played with, and whether they had access to extra calorie sources, were also linked to body condition. Because this study shows associations among customers of one food company, these findings point to questions for future research rather than proven ways to change a cat’s weight.

## 1. Introduction

Deviation from ideal body weight is a major health issue in domestic cats. The prevalence of overweight and obesity within UK-based studies ranges from 11.5% to 39% [1,2] and prevalence estimates for underweight range globally from 9.1% to 30.3% [3,4,5]. Deviations from ideal weight can be defined by a ratio over the ideal range, by body mass index (BMI), by dual-energy X-ray absorptiometry (DXA), or by computed tomography (CT) and other imaging methods; a 5- or 9-point body condition score (BCS) is the most common semi-quantitative method used in veterinary practice and epidemiological research [6]. Both excess body weight and low body weight are associated with decreased lifespan in cats [7]. Excess weight is further associated with diabetes, hypertension, arthritis, asthma, and atopic dermatitis, as well as other orthopedic, dental, skin, respiratory and urinary issues [8,9,10,11]. While low body weight is often a marker for diseases such as cancer, heart disease, and chronic gastrointestinal disease [10], the loss of lean body mass alone, particularly in older cats, impacts strength, immune function and quality of life [12].

Demographic correlates for weight status have been well-studied, but findings are not always in agreement. A major associated factor is age: middle-aged cats are more likely to be overweight [3,4,8,13,14,15,16] and senior cats are more likely to be underweight [14]. The literature also supports an association for sex, with males more likely overweight [3,8,13] and females more likely underweight [5]. Evidence on neutering is less clear: while some studies link neutering with overweight [1,15,16], others do not find it to be associated with higher odds of overweight [5,17] but rather associated with lower odds of underweight relative to ideal [5]. The literature suggests a neuter and sex interaction effect, with intact males more likely to be overweight than intact females, and neutered females more likely to be overweight than neutered males [6], though this has not been tested formally. Studies have found mixed breeds more likely to be overweight [3,8,9,14] including domestic breeds specifically [9,18]. Within the domestic category, however, medium- and long-haired cats may be more likely to be underweight [5,19]. Purebreds that have been associated with overweight status include British Shorthair [5] and Manx [9]. Those associated with non-overweight status include Burmese, Birman, and Persian [5,20,21].

Despite being more clinically actionable than demographic factors, many dietary and environmental correlates of feline obesity remain less well understood. Much of the existing evidence comes from owner-report survey studies, as these variables are rarely recorded systematically in veterinary settings. Limitations in owner-reported data reliability mean that findings in this area require replication across multiple studies before firm conclusions can be drawn, a gap the present study aims to address.

Dietary format remains a useful exposure for investigation despite nutritional heterogeneity within individual formats. Although nutrient composition expressed in g/Mcal is fundamental to dietary assessment, diet formats can also differ in moisture, physical structure, processing, and associated feeding practices. Experimental comparisons have identified differences in nutrient digestibility between diets of different formats in cats and dogs [22,23], even when processing methods were compared using similar starting formulations [24,25]. Microbiomic composition has also been demonstrated to vary by diet format [25,26,27]. These findings support investigating dietary formats as broad exposures, while recognizing that comparisons between products do not necessarily isolate processing from composition. Format-level analyses therefore complement, rather than substitute for, nutrient-level characterization.

Conventional dietary formats include extruded dry foods and retorted wet foods (henceforth dry and wet). Dry food has been associated with overweight status across multiple studies [13,14,20,28,29]. Evidence for wet food is less consistent. Because wet food is also commonly fed alongside or instead of dry food, studies reporting an association between dry food and body condition often implicitly compare higher dry-food intake with diets containing a greater proportion of wet food [1,3,20]. When examined as a distinct exposure, however, wet food has yielded null results in at least one study [14], and has been associated with increased overweight when fed between two and six months of age [29] or when offered ad libitum [15]. Differences in how wet foods are defined and grouped may contribute to this variability, with some studies using broad high-moisture categories and others distinguishing foods by packaging or product type [3,14,20,29]

Emerging formats, including commercially prepared fresh (also referred to as “mildly cooked” in the scientific literature [30,31]) foods and raw meat-based diets (also termed biologically appropriate raw food [BARF]), are less well studied in relation to body condition. Raw diets have been associated with higher odds of overweight in one study [32], while some, but not all, raw formats were associated with lower odds in another [13]. Fresh foods are distinct from raw diets because they undergo cooking, and from dry diets due to higher moisture content and lower cooking temperature [33]. Although fresh and conventional wet foods are both high-moisture formats, fresh diets are processed at lower temperatures (70–90 °C) than wet diets (115–130 °C) with corresponding differences in processing-related compounds reported in commercial products [31]. Their association with feline body condition therefore warrants further investigation.

Differences in how dietary exposure is represented may further complicate comparisons across studies. Assigning each cat to a single dietary category obscures mixed feeding, while treating individual formats as binary exposures does not capture their relative contributions. Both approaches may mask associations with the proportion of each format fed. A further limitation is that dietary associations have been examined predominantly with respect to overweight status, leaving it unclear whether factors associated with lower odds of overweight are also associated with ideal body condition or instead reflect higher odds of underweight.

Feeding schedule may also play a role, though less consistently: ad libitum feeding has been linked to overweight status in some studies [15,34,35] but not others [1,17,20,28]. Sources of additional calories, including treats, table scraps, food from neighbors, and food stolen from other household cats, have likewise been associated [13,15,28,29], although some studies found no association with treat provision [3,14]. Individual behavior shapes the balance of caloric intake and output: cats described as greedy are more likely to be overweight than those described as picky eaters [20], and increased activity is consistently associated with lower odds [3,4,17,20].

Aspects of the living environment further modulate activity level and stress, both of which influence weight. Indoor housing is frequently associated with being overweight [5,13,14,17,28], while the presence of other cats or animals in the household is associated with lower odds of being overweight [4,16]. Elevated stress, which may itself be shaped by the living environment, has also been associated with overweight status [16].

Several important gaps remain. Most studies have focused exclusively on overweight status, leaving underweight cats comparatively understudied. This is a meaningful omission, since factors associated with overweight are not simply the inverse of those associated with underweight, the two conditions warrant separate investigation [5]. Equally neglected in prior research is the question of what factors are associated with ideal body condition. Several mechanisms may push body condition in either direction depending on the individual cat: some dietary formats may be associated with more stable energy intake or feeding patterns, potentially reducing weight deviations; stress responses may drive some cats toward over- and others toward undereating; dietary components such as probiotics may exert a normalizing effect; and multi-cat households introduce dynamics that are difficult to predict at the individual level. Modeling ideal body condition as a distinct outcome, rather than inferring it from the overweight and underweight models, allows these hypothesized stabilizing correlates to be examined directly.

The present study examines two related questions relevant to the assessment and management of feline body condition. The first asks which modifiable feeding and environmental factors are associated with weight. To address it, we use a broad owner-reported survey capturing a wide range of demographic and lifestyle factors hypothesized to be associated with general cat health, with particular emphasis on factors that are readily modifiable by cat owners. A question of particular interest was whether a greater dietary contribution from fresh food was associated with ideal body condition. We investigated this question within a broader exploratory analysis of dietary formats and other feeding and environmental characteristics. Overweight, underweight, and ideal body condition were examined separately to characterize the pattern of associations across these categories. Because dietary contributions are interdependent, any association with fresh food was interpreted relative to the other formats comprising the diet, rather than as an isolated effect. Given the inconsistent findings around treats in the literature, we further distinguish between specific treat types, asking sufficiently detailed questions to begin resolving prior conflicting reports on additional calorie sources. Together, these variables allow for a more granular characterization of dietary associations than has been possible in prior work. As demographic characteristics are well-established correlates of BCS, we retain these variables in all models as a priori adjustment factors, rather than as exposures to be interpreted on statistical grounds; we therefore additionally fit a demographics-only model for each outcome, to provide total demographic associations, including those that may operate through downstream feeding and environmental variables. Given the breadth of factors examined and the cross-sectional design, the analysis was exploratory and intended to identify candidates for prospective evaluation rather than to test prespecified causal hypotheses.

Owner-reported data allow more direct knowledge of their cats’ behavior in their usual ecological context and, although direct owner recruitment introduces selection and reporting biases, it avoids biases associated with veterinary presentation, clinic-specific diagnostic practices, and what clinicians choose to record or code, while structured questionnaires may elicit more accurate owner-reported information than unprompted veterinary consultations [36]. We complement the primary analysis with robustness checks of perception bias and differential owner engagement: we test whether the diet associations are sensitive to systematic differences in how owners perceive or report body condition across food types, and whether owners feeding different diets differ in their reported weight-management behavior.

The second aim asks whether presenting owners with a structured BCS tool shifts body weight classification. This question is equally consequential for practice, because intervention depends on owner recognition. We hypothesize that owners have some capacity to perceive weight deviation even in the absence of formal guidance, but that this unstructured perception meaningfully underestimates the prevalence of weight problems and can be shifted when owners are given structured tools. To test this, we compare owner-reported weight assessments made without guidance against assessments anchored to objective body condition descriptions, and hypothesize that fewer cats will be identified as ideal weight under the structured condition. We further predict that among cats in abnormal weight categories, those whose owners’ unguided assessment is in agreement with the guided assessment will be more likely to have received dietary and lifestyle interventions.

## 2. Materials and Methods

### 2.1. Data Acquisition and Cleaning

#### 2.1.1. Study Population

The study population comprised pet cats owned by current and prospective customers of a direct-to-consumer UK company producing fresh cat food. Between January and July 2025, owners of cats either already receiving the subscription diet or newly enrolled but not yet transitioned to it were emailed an invitation to participate in a cat health study by completing two surveys: a demographic survey, which captured signalment, birth history, and acquisition source, and a weight survey, which captured feeding practices, activity, household characteristics, and body condition status. In addition, owners reported any diagnosed medical conditions in their cat, allowing basic characterization of general health status. Question and response wordings are detailed in Table S1. The surveys were distributed only to customers with a single subscribed cat. As some households contained additional cats that were not receiving the subscription diet, duplicate households were removed to ensure that only one cat per household contributed survey data. Following sample size precedents calculated from similar studies in humans [13], potential participants were contacted in randomly selected segments of 2000–5000 until achieving a sample size of over 5500.

#### 2.1.2. Body Condition Status

Survey participants were presented with visual and written descriptions of a 9-point BCS scale adapted from WSAVA [37] with weight classifications (underweight, ideal, overweight) removed to reduce bias (Figure S1). Written descriptions were added as the inclusion of written descriptions has also been shown to significantly increase owner agreement with veterinarian assessment [38]. Answers were collapsed into three categories for all subsequent analysis: underweight (BCS 1–3), ideal weight (BCS 4–5), and overweight and obese (henceforth “overweight”) (BCS 6–9). BCS 6 was classified as overweight in accordance with the conventional WSAVA threshold [37]; however, as BCS 6 has previously been included within the ideal category [5], we repeated the variable-selection procedure and subsequent multivariable modeling with this alternative grouping as a sensitivity analysis. To improve readability throughout the manuscript, the labels “underweight,” “ideal,” and “overweight” denote categories derived from owner-assigned BCS.

#### 2.1.3. Unguided Owner-Perceived Weight Status

In addition to visual and written descriptions, survey participants were asked to indicate whether they perceived their cat as overweight or underweight (yes, no, or unsure). Unlike BCS, which is anchored to standardized visual and descriptive criteria, this measure reflects a more subjective assessment of body condition. It therefore captures owner perception directly and is used as a sensitivity analysis to assess potential bias in BCS perception. Throughout the manuscript, this classification will be denoted as “unguided owner perception.”

#### 2.1.4. Owner Weight-Control Behavior

Survey participants were asked whether they had ever attempted to control their cat’s weight (yes/no). This measure reflects owner engagement in weight management and provides an indicator of whether weight is perceived as a concern. It was used in a sensitivity analysis to assess whether behavior directed toward weight control differed across food types and therefore whether differences in this aspect of owner behavior could contribute to observed associations.

#### 2.1.5. Age

Age was calculated from reported year and month of birth. For cats missing month of birth (*n* = 302), age was estimated assuming midyear (June). Cats with missing birth year were excluded from analysis. Because exploratory analyses indicated non-linear associations between age and BCS, age was modeled as a categorical variable with the following groupings [39,40]:

- Kitten: birth to <1 year;
- Young adult: 1–<7 years;
- Mature adult: 7–<10 years;
- Senior: 10–<15 years;
- Geriatric: ≥15 years.

Given the reported lack of validation of body condition scoring for assessing body composition in kittens [41], we conducted a sensitivity analysis excluding cats classified as kittens. The variable-selection procedure and subsequent multivariable modeling were repeated separately for the overweight, underweight, and ideal-body-condition outcomes.

#### 2.1.6. Breed

Breeds with more than 200 individuals were retained as separate categorical levels. Domestic short-, medium-, and long-haired cats were coded as distinct categories due to hypothesized differences in BCS estimation associated with hair length.

Other purebred cats were grouped according to shared ancestry [42,43] into broader breed groups (Western, Asian, Mediterranean, and Hybrid) (Table S2). Cats reported as “Moggies,” “Mixed,” or identified by owners as having mixed ancestry were categorized as mixed-breed.

#### 2.1.7. Sex and Neuter Status

Sex and neuter status were included as separate variables, and their interaction was explicitly modeled. This decision was based on the established literature indicating differential metabolic and behavioral effects of neutering by sex with etiological rationale [44].

#### 2.1.8. Diet Composition

Owners reported current diet composition across food format (dry, wet, fresh, raw, and “other” food formats) using a two-step approach. First, respondents indicated whether each diet format was fed (yes/no). For endorsed formats, they were asked what portion of the cat’s regular meal calories came from that diet type, accompanied by descriptive anchors to aid interpretation (0–10%: a negligible amount; 10–40%: some; 40–60%: about half; 60–90%: most; 90–100%: almost all; or “unknown”). These non-equidistant intervals were selected to align with common vernacular descriptions of proportion, balancing interpretability and response accuracy while acknowledging inherent uncertainty in estimating dietary composition within certain ranges. Missing values in the dietary composition variables arising from the owner endorsing the diet type but selecting “unknown” for the percentage were imputed using rule-based deterministic procedures. Where only one diet type was endorsed, the missing value was set to 90–100%. In rows with exactly one missing value, one non-zero value, and the remaining categories not endorsed, the missing value was imputed as the complement to 100 (i.e., 100 minus the observed range). Because univariable analyses suggested approximately monotonic trends, percentage categories were converted to numeric proportions using the midpoint of each reported range (e.g., 40–60% encoded as 0.50). These were entered as continuous proportion variables in multivariable models. This approach preserves greater resolution than classifying cats into a single dominant diet category or coding each diet format as binary (present/absent) and allows assessment of graded/trend associations. Reported ORs correspond to a one-unit (0 to 1) increase in the proportion and primarily quantify the direction and strength of the modeled trend across increasing dietary contribution. Because dietary proportions are compositional and not all formats were retained in every model, these ORs should be interpreted relative to the combined contribution of formats omitted from that model, conditional on any other retained dietary proportions.

#### 2.1.9. Outdoor Access

Multiple descriptive answer options related to outdoor exposure (Table S3) were consolidated into two categorical variables:

- Maximum type of outdoor space accessible;
- Presence or absence of unsupervised outdoor access.

This consolidation reduced dimensionality while preserving meaningful distinctions in environmental exposure.

#### 2.1.10. Household Composition

Numeric variables representing numbers of cats, dogs, children, adults, and rooms in the household were truncated at upper limits (4, 3, 3, 4, and 10 respectively) to reduce the influence of extreme values and improve model stability.

#### 2.1.11. Ordinal Behavioral Variables

Ordinal variables (stress frequency, observed water intake frequency, play frequency, and play duration) were collapsed into the broader categorical groupings presented in the tables prior to analysis. This decision was based on exploratory analyses indicating non-linear and non-monotonic relationships with BCS.

### 2.2. Statistical Analysis

Continuous variables are presented as mean ± SD. Categorical and ordinal variables are presented as count or % of total count. Unless stated otherwise, *p* < 0.05 was considered statistically significant. No adjustment for multiple comparisons was applied to the reported odds ratios, confidence intervals, or *p*-values. Multiplicity procedures such as the Bonferroni or Benjamini–Hochberg methods require a prespecified family of hypotheses. Because the number and choice of tests depended on the data in the present analysis, there is no principled way to define the family of tests to which a correction should apply. Consistent with recommendations for exploratory analyses, results are therefore reported without multiplicity adjustment and are explicitly labeled as exploratory [45]. All analyses were performed in R version 4.3.2.

#### 2.2.1. Multiple Imputation

Eighteen variables contained missing data, arising from either selection of an “unknown” response option or incomplete survey responses, with missingness ranging from 0.34% to 10.3% (Table S4). These missing data were addressed using multiple imputation by chained equations (MICE) [46]. Five imputed datasets were generated using fully conditional specification, with 30 iterations per imputation to ensure convergence of the imputation models. All variables considered in the analysis, including the outcome, candidate predictors, and prespecified covariates, were included in the imputation models to preserve associations among variables and reduce bias attributable to missingness. Continuous variables were imputed using predictive mean matching using a donor pool of five observations, and categorical variables were imputed using logistic or multinomial regression as appropriate. All subsequent modeling steps were performed separately within each imputed dataset. Model estimates were combined across imputations using Rubin’s rules [47] to account for within- and between-imputation variability.

#### 2.2.2. Variable Selection Strategy

A structured, purposeful selection strategy was used to identify predictors of weight status. This approach was selected over automated backward stepwise regression because it better preserves confounding control, reduces model instability, and prioritizes interpretability and epidemiologic validity [48]. Each body condition category (overweight, underweight, and ideal) was modeled as a separate binary outcome rather than fitting a single multinomial model. First, purposeful selection allowed each model to retain a condition-specific set of predictors, consistent with our premise that the correlates of overweight and underweight are not simply mirror images of one another; a multinomial model would have constrained both contrasts to share a single predictor set. Second, separate binary models are more robust to the owner-reported misclassification expected near adjacent BCS boundaries, since each contrast does not depend on the precise allocation of cats across all three categories simultaneously. Third, the resulting odds ratios, each expressed relative to all other cats, are more directly interpretable than two simultaneous contrasts against a common reference. To confirm that our conclusions did not depend on this choice, we additionally fitted a multinomial logistic regression model with ideal body condition as the reference category using the same variable-selection protocol.

#### 2.2.3. Demographic Covariates (Forced Variables)

A priori covariates were retained in all models regardless of statistical significance. These included breed, age group, sex, neuter status, and the sex–neuter interaction. These variables were selected based on biological plausibility and prior evidence linking them to body condition and potential confounding of other exposures. Forcing these variables into all models ensures that estimated associations for other predictors are adjusted for key demographic and physiologic factors, preventing residual confounding and improving interpretability.

Because feeding, activity, and household variables may lie on the causal pathway between demographic factors and body condition (e.g., neuter status may influence weight partly through changes in appetite or activity), the demographic coefficients reported within the primary models are conditional on these downstream variables and should not be interpreted as estimates of total demographic effects [49]. To provide estimates less likely to be attenuated by mediation, we additionally fit a demographics-only model for each outcome, containing only breed, age group, sex, neuter status, and the sex–neuter interaction, with no feeding, activity, or household covariates. Coefficients from this model are more directly interpretable as overall associations between demographic factors and body condition; coefficients from the full primary models should instead be read as associations conditional on the exposures also included.

#### 2.2.4. Univariable Screening

Candidate predictors were first evaluated in univariable logistic regression models. A liberal screening threshold of 0.25 was chosen to avoid prematurely excluding variables that may become important after confounder adjustment, as strict univariable thresholds can miss predictors that are confounded or suppressed by other variables [48].

#### 2.2.5. Multivariable Model Construction

An initial multivariable model was constructed including all prespecified covariates and screened candidate predictors. Backward elimination was then performed using likelihood ratio tests with a stay criterion of α = 0.10 [48]. At each step, the least statistically significant variable was considered for removal.

#### 2.2.6. Change-in-Estimate (Confounding) Assessment

Before removing a variable, its impact on key parameter estimates was evaluated. Specifically, the percent change in magnitude of coefficients for prespecified target variables (demographic covariates) was calculated. If removal of a candidate variable altered any target coefficient by more than 15%, the variable was retained as a confounder, even if not statistically significant [50].

#### 2.2.7. Add-Back Procedure

Following backward elimination, excluded variables were re-evaluated individually in the reduced model. Variables with a likelihood ratio test *p*-value ≤ 0.15 were reintroduced [48]. This add-back step reduces the risk of excluding variables that may have become important after removal of correlated predictors and improves model stability.

#### 2.2.8. Stability Assessment

To evaluate robustness of variable selection, bootstrap resampling was performed within each imputed dataset. For each resample, the purposeful selection procedure was repeated, and inclusion frequencies were calculated for candidate predictors. To be included in the final parsimonious model, a candidate predictor was required to be retained in 100% of bootstrap resamples within each imputed dataset, reducing the likelihood of reporting sample-dependent associations and protecting against overfitting.

#### 2.2.9. Diagnostics

Model diagnostics were assessed within each imputed dataset. Multicollinearity was evaluated using variance inflation factors (VIFs), with adjusted generalized VIFs calculated for categorical predictors GVIF^(1/(2 × df)) [51]. Influential observations were assessed using Cook’s distance [52]. Potential separation was evaluated using bias-detection diagnostics applied to the model matrix (detectseparation package version 0.4.0) [53]. Model calibration was assessed using the Hosmer–Lemeshow goodness-of-fit test [54].

## 3. Results

### 3.1. Cohort Characteristics

Between January and July 2025, a total of 29,237 cat owners in the UK were contacted and invited to participate in a health assessment-based study, resulting in 5653 completed surveys. Cats included were aged 8.13 ± 5.44 years, 48.7% female, and 95% neutered. Approximately 69% of cats were non-pedigree, comprising Domestic Shorthairs (37%), Domestic Medium (7%) and Long (5%) Hairs, and mixed-breed cats (20%); the remaining 31% were owner-reported purebreds. Regarding primary diet composition, 81% received at least some fresh food, with 47% receiving multiple types. Owner-assessed BCS scores were distributed across the full 9-point scale. Overall, 20% of cats were classified as underweight based on scores 1–3, including 95 cats with a score of 1 (2%), 245 with a score of 2 (4%), and 794 with a score of 3 (14%). Just over half of cats (53%) were classified as ideal weight based on scores 4–5, comprising 762 cats with a score of 4 (13%) and 2215 with a score of 5 (39%). The remaining 27% were classified as overweight or obese based on scores 6–9, including 695 cats with a score of 6 (12%), 690 with a score of 7 (12%), 134 with a score of 8 (2%), and 23 with a score of 9 (<1%). These classifications were carried through for all subsequent analyses. Full cohort characteristics are reported in Table S5. Medical issues were reported for 36% of cats, with dental issues (11%), other (8%), and allergies (5%) as the most commonly endorsed conditions (Table S6); however, the majority of cats were not reported by their owners to have a diagnosed condition.

### 3.2. Unguided Owner Weight Perception and Intervention

Of the full dataset of 5653 cats, 5552 had complete owner perception data. In this subset, 27% of cats were classified as overweight, 20% as underweight, and 53% as ideal by weight class derived from owner-assessed BCS using a structured tool. In contrast, using unguided owner perception, 14% were classified as overweight, 10% as underweight, 62% as ideal, and 15% as unsure. The cross-classification of these measures is presented in Table 1. Agreement between the two measures varied by category: among cats classified as ideal by BCS, 77% were also perceived as ideal; among those classified as overweight by BCS, 43% were perceived as overweight; and among those classified as underweight by BCS, 30% were perceived as underweight. After excluding “unsure” responses, 68% of classifiable cases had matching BCS and unguided classifications. The unweighted kappa was 0.418, while the linear and quadratic weighted kappas were 0.469 and 0.545, respectively.

Overall, in the perception subgroup, 53% of owners reported attempting to control their cat’s weight, while 47% reported no such attempts. Within BCS categories, owner perception was strongly associated with reported weight control behavior. Among cats classified as overweight by BCS, 91% of owners who perceived their cat as overweight reported attempting weight control, compared to 54% of those who perceived their cat as ideal. Similarly, among cats classified as underweight by BCS, 71% of owners who perceived their cat as underweight reported attempting weight control, compared to 28% of those who perceived their cat as ideal. Among cats with both ideal BCS and an unguided perception of ideal weight, 40% of owners reported attempting to control their cat’s weight.

### 3.3. Univariable Analysis

Of 58 candidate variables, 32 were significantly associated with overweight status at *p* < 0.05, notably age group, breed, neuter status (OR 4.38), dry food proportion (positively associated) and wet food proportion (inversely associated), and several additional-calorie-source variables including household members, neighbors, other cats’ food, stolen food, and table scraps. For underweight, 34 variables reached significance, including fresh food proportion (inversely associated) and neuter status (OR 0.53). For ideal weight, 23 variables were significant, including an increasing positive association with fresh food proportion and several enrichment variables (cat tower, scratching post, string/wand toy, tunnels, and window seat). Full univariable associations are presented in Table S5.

### 3.4. Demographics-Only Models

Age, breed, sex, neutering, and the sex–neuter interaction were associated with weight status. Kittens tended toward lower body condition, whereas mature adults, seniors, and geriatric cats were less likely to be ideal weight, with patterns differing across overweight and underweight outcomes. Several breed groups also differed from Domestic Shorthairs. Male and neutered cats were less likely to be underweight and more likely to be ideal weight, with significant sex-by-neuter interactions. Full results for the demographics-only models are in Table 2.

### 3.5. Final Overweight Model

**Figure 1:**
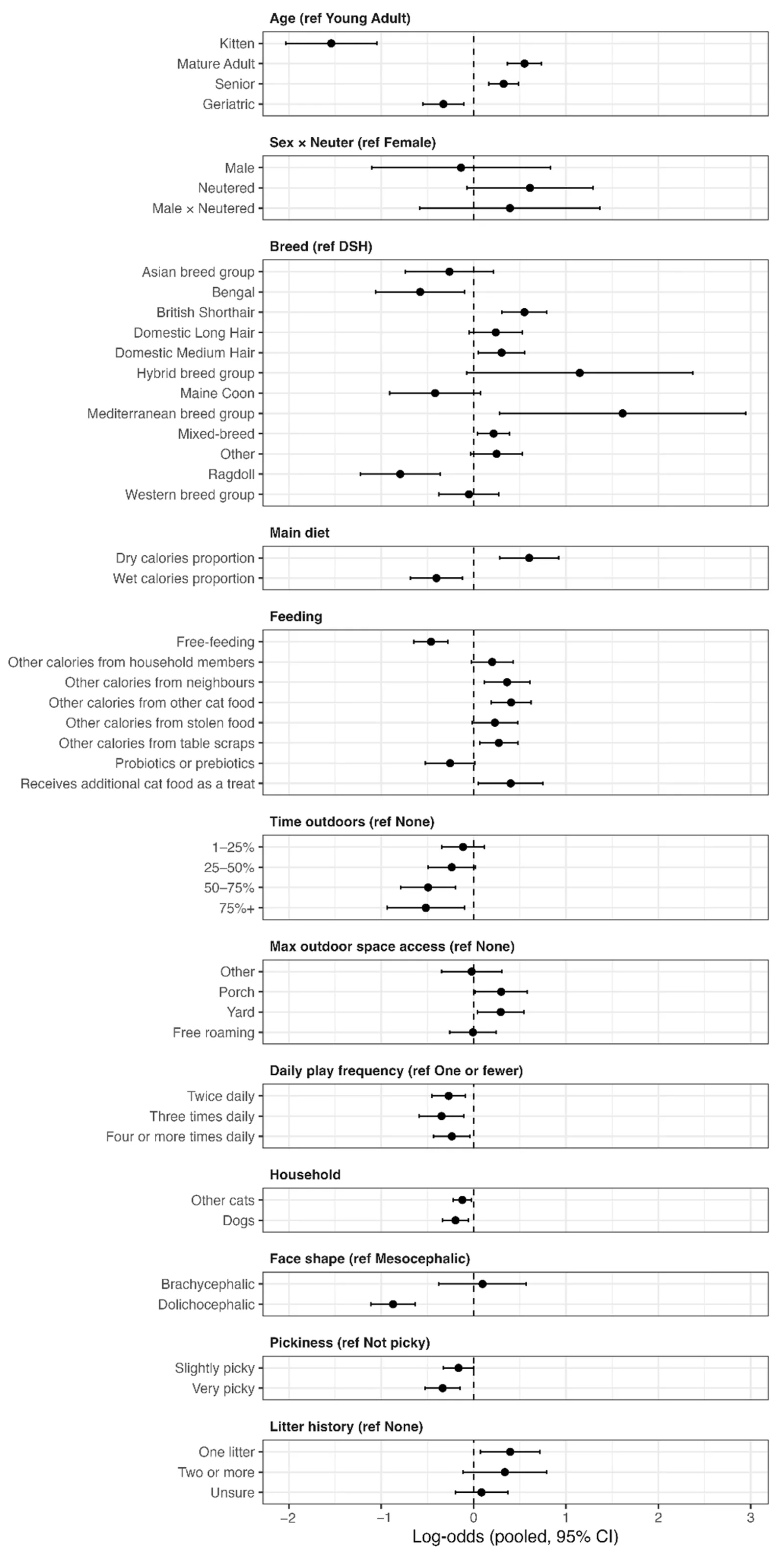
Multivariable regression coefficients for factors associated with overweight body condition in cats.

Including the five forced demographic predictors, 23 of 58 predictors were retained in the final overweight model (Table 3, Figure 1). Variable inclusion frequencies are in Table S7A.

Forest plots show log-odds regression coefficients and 95% confidence intervals (CIs) from the final multivariable logistic regression model comparing overweight versus non-overweight cats. Points represent estimated regression coefficients and horizontal bars represent 95% CIs. The dashed vertical line at zero indicates no association with overweight status. Positive coefficients indicate increased odds of being overweight, whereas negative coefficients indicate reduced odds relative to the reference category. Reference groups for categorical predictors are indicated in panel headings. Age group, breed, sex, neuter status, and the sex–neuter interaction were forced a priori as adjustment variables and should not be interpreted as total demographic associations.

Feeding. A higher proportion of calories from dry food was associated with greater odds of overweight, whereas a higher proportion from wet food and free feeding were associated with lower odds. Feeding by neighbors, eating other cats’ food, table scraps, and additional cat food treats were associated with greater odds of overweight. Greater reported pickiness was associated with lower odds. Feeding by other household members, stealing food, and probiotic/prebiotic supplementation were retained but were not statistically significant.

Environment. Cats whose maximum permitted outdoor access was a porch or yard had greater odds of overweight than cats with no outdoor access, whereas greater reported time outdoors was associated with lower odds. More frequent play and increasing numbers of cats and dogs in the household were also associated with lower odds. Other outdoor-access categories were not significant.

Demographic. Age and breed associations were broadly consistent with the demographics-only model. After adjustment, Domestic Medium Hair, Mediterranean-group, mixed-breed, and Ragdoll cats also differed significantly from Domestic Shorthairs. Sex, neuter status, and their interaction were not significantly associated with overweight. Dolichocephalic cats had lower odds of overweight than mesocephalic cats, and cats with one litter had higher odds than cats with no litters or males.

### 3.6. Final Underweight Model

**Figure 2:**
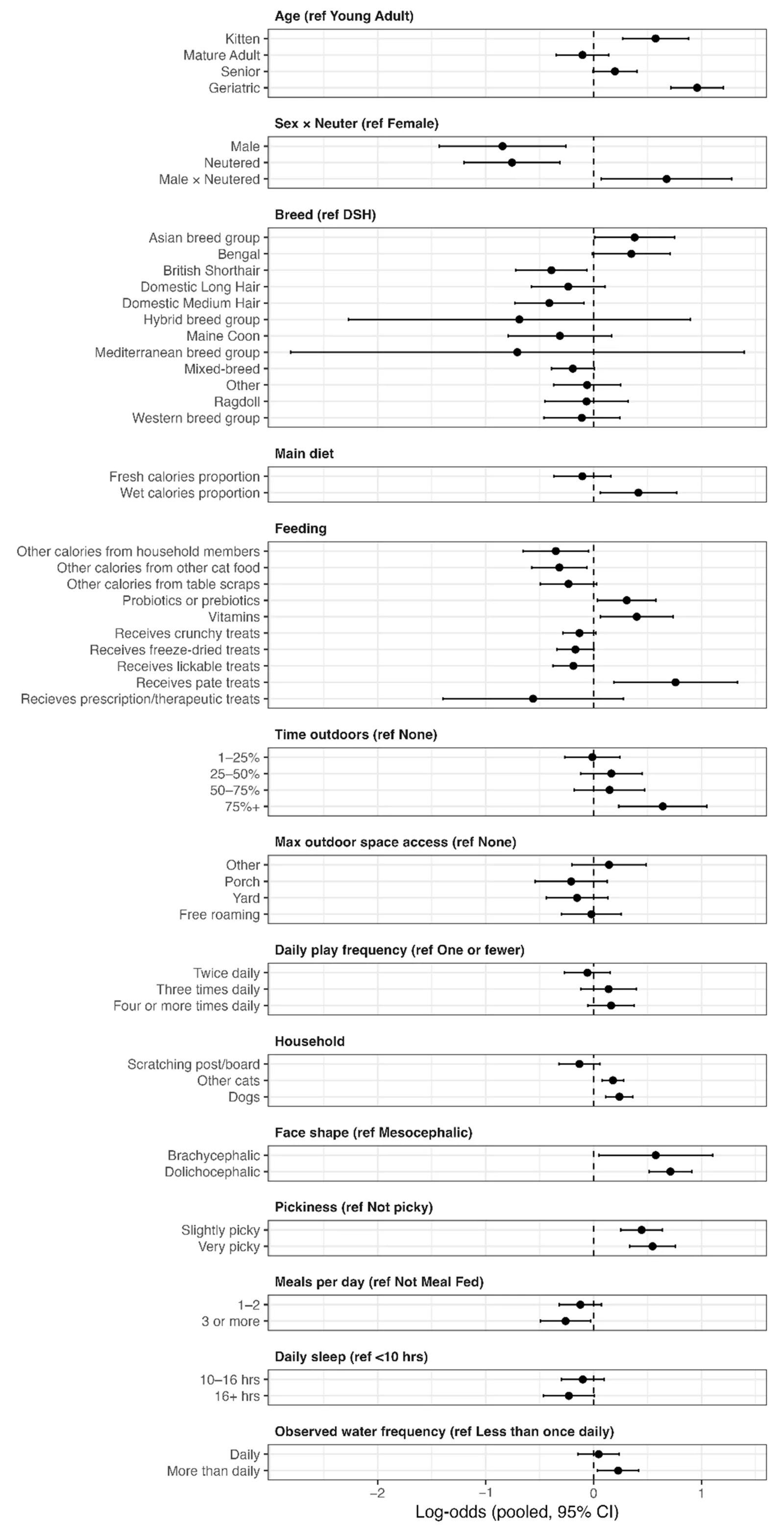
Multivariable regression coefficients for factors associated with underweight body condition in cats.

Including the five forced demographic predictors, 28 of 58 predictors were retained in the final underweight model (Table 4, Figure 2). Variable inclusion frequencies are in Table S7B.

Forest plot showing log-odds regression coefficients and 95% confidence intervals (CIs) from the final multivariable logistic regression model comparing underweight versus non-underweight cats. Points represent estimated regression coefficients and horizontal bars represent 95% CIs. The dashed vertical line at zero indicates no association with underweight status. Positive coefficients indicate increased odds of being underweight, whereas negative coefficients indicate reduced odds relative to the reference category. Reference groups for categorical predictors are indicated in panel headings. Age group, breed, sex, neuter status, and the sex–neuter interaction were forced a priori as adjustment variables and should not be interpreted as total demographic associations.

Feeding. A higher proportion of calories from wet food, pâté treats, increasing pickiness, and probiotic/prebiotic or vitamin supplementation were associated with greater odds of underweight. Feeding by other household members, eating other cats’ food, and receiving three or more meals per day were associated with lower odds. Several additional feeding variables were retained but were not statistically significant.

Environment. Spending at least 75% of time outdoors, frequent observed water consumption, and increasing numbers of cats and dogs in the household were associated with greater odds of underweight. Scratching-post access, sleep, play frequency, and maximum outdoor-space access were retained but were not statistically significant.

Demographic. Most demographic associations were consistent with the demographics-only model. After adjustment, British Shorthair and Domestic Medium Hair cats had lower odds of underweight, while several breed associations observed in the demographic-only model were no longer significant. Male sex, neutering, and their interaction remained significant. Brachycephalic and dolichocephalic cats had greater odds of underweight than mesocephalic cats.

### 3.7. Final Ideal Weight Model

**Figure 3:**
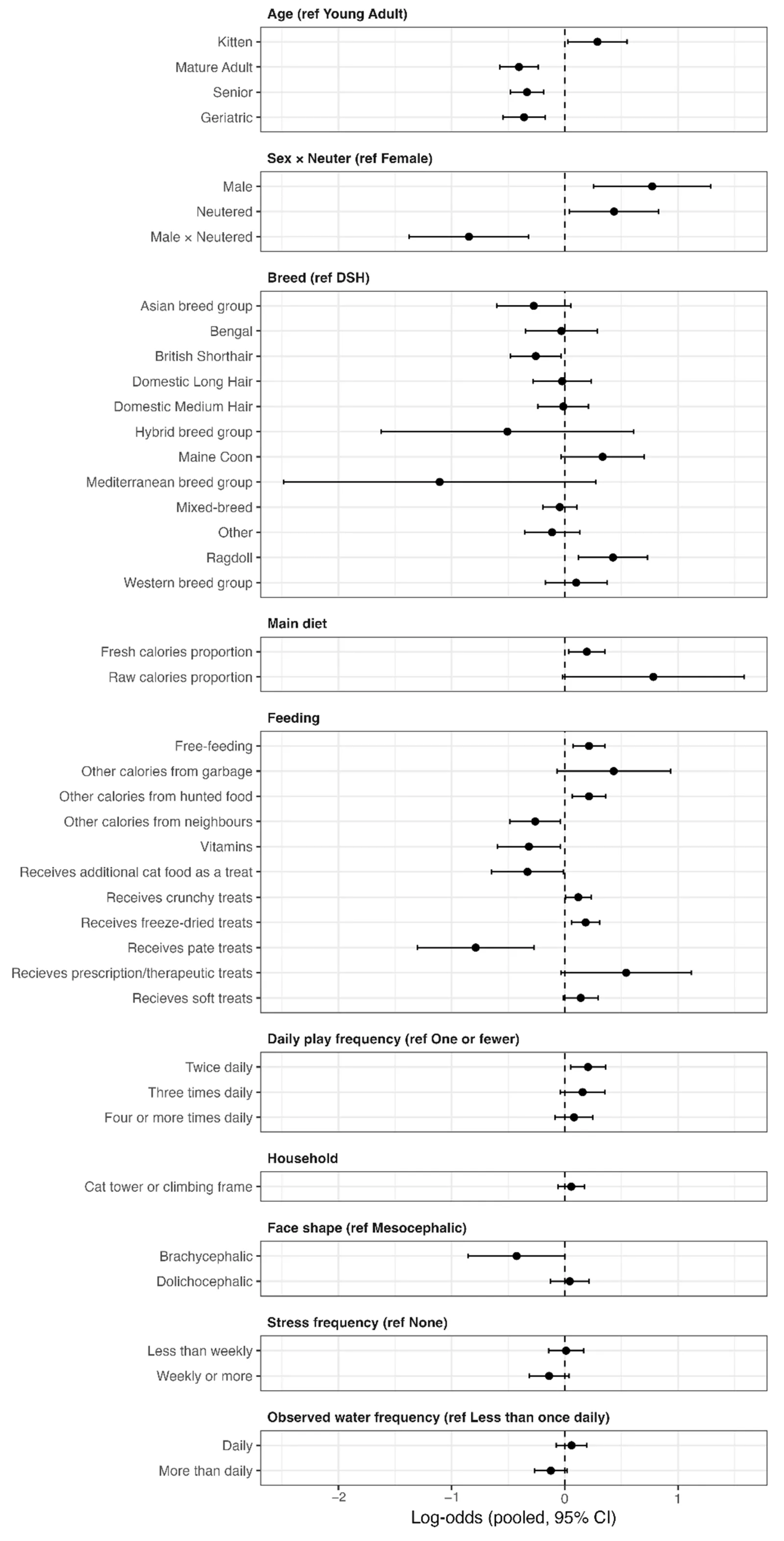
Multivariable regression coefficients for factors associated with ideal body condition in cats.

Including the five forced demographic predictors, 23 of 58 predictors were retained in the final ideal-weight model (Table 5, Figure 3). Variable inclusion frequencies are in Table S7C.

Forest plots show log-odds regression coefficients and 95% confidence intervals (CIs) from the final multivariable logistic regression model comparing ideal weight versus non-ideal weight cats. Points represent estimated regression coefficients and horizontal bars represent 95% CIs. The dashed vertical line at zero indicates no association with ideal status. Positive coefficients indicate increased odds of being ideal weight, whereas negative coefficients indicate reduced odds relative to the reference category. Reference groups for categorical predictors are indicated in panel headings. Age group, breed, sex, neuter status, and the sex–neuter interaction were forced a priori as adjustment variables and should not be interpreted as total demographic associations.

Feeding. A higher proportion of calories from fresh food, free feeding, hunting food, and crunchy or freeze-dried treats were associated with greater odds of ideal weight. Additional cat food treats, pâté treats, feeding by neighbors, and vitamin supplementation were associated with lower odds. Other retained feeding variables were not statistically significant.

Environment. Being played with twice daily was associated with greater odds of ideal weight. Other retained environmental factors, including stress frequency, observed water consumption, and access to a cat tower or climbing frame, were not statistically significant.

Demographic. Age, sex, neutering, and the sex-by-neuter interaction remained broadly consistent with the demographics-only model. After adjustment, British Shorthair cats had lower odds and Ragdoll cats had higher odds of ideal weight relative to Domestic Shorthairs. Other breed and craniofacial morphology differences were not statistically significant.

### 3.8. Results of Model Diagnostics

All VIFs other than those for sex–neuter interaction terms were below 2, indicating no evidence of problematic multicollinearity. The maximum Cook’s distance across imputations was 0.0069, 0.021, and 0.0081 for the overweight, underweight, and ideal weight models respectively, indicating that no single observation exerted undue influence on model estimates. No evidence of separation was detected. The overweight and underweight models showed no evidence of lack of fit across imputations (minimum *p*-values 0.16 and 0.08, respectively). For the ideal weight model, most Hosmer–Lemeshow tests were non-significant, although occasional significant results were observed (minimum *p* = 0.02); given the sensitivity of this test to large sample sizes and the absence of other diagnostic concerns, overall calibration was considered acceptable. Full diagnostic results are provided in Table S8.

### 3.9. Robustness Checks

#### 3.9.1. Owner Perception and Body Condition

We tested whether owner perception of weight varied by food type, to assess whether the observed food type associations may be biased by differential misperceptions across diet. Two owner-reported adiposity measures were collected: (1) BCS-derived weight status, based on owner assignment using pictorial and verbal descriptors (with weight class labels removed), and (2) unguided owner perception. The sample was restricted to cats receiving 90–100% of their caloric intake from a single diet type, leaving a sample of 2026 cats. We then fitted three logistic regression models, for overweight, underweight, and ideal weight, with unguided owner perception as the outcomes, including food type, BCS-derived weight status, their interaction, and survey age group as a covariate. Type III likelihood ratio tests were used to assess the overall significance of model terms.

For overweight there was no evidence that the relationship between BCS status and unguided owner perception overweight differed by food type (food type × BCS interaction: χ^2^ = 0.78, df = 2, *p* = 0.68), and the same holds true for underweight (food type × BCS interaction: χ^2^ = 1.6, df = 2, *p* = 0.44) as well as ideal weight (food type × BCS interaction: χ^2^ = 2.7, df = 2, *p* = 0.26). Results are listed in Table S9.

#### 3.9.2. Owner Weight Control Behavior

We examined whether owner-reported attempts to control their cat’s weight differed by food type using a binary indicator of weight control behavior. We adjusted for BCS-derived body condition status, as owners are more likely to initiate weight control efforts in response to their cat’s current weight, making body condition a key determinant of this behavior. We also adjusted for age group, as older cats have had more opportunity for weight management interventions over time.

Overall, 56% of owners reported attempts to control their cat’s weight, and 44% reported that they did not make these attempts. There was no evidence that owner-reported weight control behavior differed by food type (Type III test: χ^2^ = 4.23, df = 2, *p* = 0.12). In contrast, weight control behavior was strongly associated with body condition, with owners of overweight cats substantially more likely to report attempting weight control (Table S9).

#### 3.9.3. BCS 6 Coded as Ideal

We classified a BCS of 6 as overweight, whereas some prior studies have grouped it with the ideal category [5]. To assess whether this choice influenced our findings, we repeated the full variable-selection protocol (univariable screening, backward elimination with change-in-estimate confounding checks, and bootstrap stability assessment) with BCS 6 reclassified as ideal. With regard to the dietary findings, the final overweight and ideal models were largely consistent with the primary analysis, both in the variables retained and in the direction and magnitude of their associations (Table S10). In particular, the three principal dietary findings persisted: a higher proportion of dry food remained associated with overweight, a higher proportion of wet food with reduced odds of overweight, and a higher proportion of fresh food with ideal condition, all retained through selection and remaining statistically significant.

#### 3.9.4. Analysis in Adult Cats Only

As BCS scoring has not been validated for kittens [41], we repeated the full variable-selection protocol (univariable screening, backward elimination with change-in-estimate confounding checks, and bootstrap stability assessment) excluding cats under one year of age. The principal dietary associations in the overweight and ideal-body-condition models were consistent with those observed in the primary analysis: higher owner-estimated proportions of meal calories from dry food were associated with higher odds of overweight, higher proportions from wet food with lower odds of overweight, and higher proportions from fresh food with higher odds of ideal body condition (Table S11). In the adult-only underweight model, the positive association with wet-food proportion no longer reached statistical significance, whereas fresh-food proportion was significantly associated with lower odds of underweight. Dry-food proportion was additionally retained in this model and was also associated with lower odds of underweight.

#### 3.9.5. Multinomial Model

Although we fitted three separate binary models for the reasons outlined above, a single multinomial model is an alternative specification for a three-level outcome. To confirm that our conclusions did not depend on this choice, we repeated the variable-selection protocol (univariable screening, backward elimination with change-in-estimate confounding checks, and bootstrap stability assessment) using a multinomial model with ideal body condition as the reference category. The resulting associations were broadly consistent with those from the separate binary models in direction and magnitude (Table S12). Because ideal serves as the reference, odds ratios for ideal body condition are not estimated directly; however, a higher proportion of fresh food was associated with significantly lower odds of underweight, and overweight was significantly associated with a higher proportion of dry food and a lower proportion of wet food.

## 4. Discussion

This study identified multiple factors associated with overweight, underweight, and ideal body condition in companion cats. To our knowledge, it is also among the first to examine correlates of ideal weight as a distinct outcome rather than treating ideal status simply as the inverse of separate overweight and underweight models. We modeled each category as a separate binary outcome rather than as a single multinomial model so that each could retain a condition-specific predictor set and remain robust to misclassification at adjacent BCS boundaries; a multinomial model fitted as a sensitivity analysis yielded associations consistent in direction and magnitude with the separate models. To complement this approach, we also modeled overweight against underweight/ideal combined and underweight against overweight/ideal combined. This more conservative framing was intended to reduce the impact of uncertainty in owner classification of discrete weight categories while still identifying correlates of relatively higher and lower body weight. Taken together, these analyses yielded several novel associations, including potentially modifiable associations involving diet format, feeding practices, and household environment. The study also provides insight into owner perception of feline weight, which may be relevant to veterinarians seeking to improve recognition and management of abnormal body condition.

By owner-assessed BCS, 27% of cats were classified as overweight or obese. This estimate is comparable to a recent UK study in which 29% were classified as such [19]. The prevalence of underweight cats in the present sample (20.1%) was within the range reported previously from 9.1% to 30.3% [3,4,5]. The distribution of owner-assigned scores may be influenced by how respondents interpret the visual or written descriptions, and therefore may reflect reporting influences. In addition to BCS, we collected owners’ unguided assessments of their cats’ weight status. A substantial proportion (15%) of owners reported uncertainty, which itself represents a practical opportunity for education. More importantly, discordance between unguided perception and the more standardized BCS-based assessment suggests that some owners are confident but inconsistent in their judgments. Consistent with our hypothesis, owners were generally more willing or able to identify overweight or underweight when given a structured BCS tool than when asked directly to classify their cat’s weight. Because the BCS instrument provided pictorial and descriptive anchors, and because weight-class labels were removed, it may have reduced both ambiguity and potential social desirability bias.

Within abnormal BCS categories, owners whose unguided classification aligned with the structured BCS classification were more likely to report implementing weight-control measures, as we hypothesized. This pattern is consistent with the idea that recognition of abnormal body condition is a prerequisite for action, although the cross-sectional design does not allow us to determine whether recognition preceded intervention or whether prior intervention increased owner awareness. Recognition of underweight (30% concordantly identified as underweight) appeared poorer than recognition of overweight (43% concordantly identified as overweight) and less likely to be addressed when recognized (71% vs. 91%), which may reflect the greater familiarity of owners with messaging around overweight than underweight. Because low body condition can signal underlying disease yet attracts less public messaging, this argues for clinicians proactively raising underweight rather than waiting for owners to volunteer concern. Interestingly, a substantial proportion of cats classified as ideal by both measures were nevertheless reported to be subject to weight-control efforts, suggesting that ideal body condition may be maintained through active owner management.

Feeding practices showed consistent associations with body condition. We identified a modest novel association between fresh food and ideal weight. This association remained statistically significant when BCS 6 was reclassified as ideal and after excluding kittens. Under the multinomial specification, fresh-food proportion was associated with lower odds of underweight relative to ideal body condition, and a negative association with underweight was also observed in the adult-only underweight model. Together with the direction of estimates in univariable analysis, this pattern suggests that the fresh-food association with ideal body condition may reflect lower odds of underweight more than lower odds of overweight. Potential biological mechanisms underlying associations with fresh diets include variation in macronutrient composition, moisture content, palatability, and digestibility [22]. Diets characterized by higher protein and lower carbohydrate content have previously been shown to alter both feline gut microbiome and metabolite profiles [55,56], which may subsequently influence satiety signaling. The high prevalence and wide range of fresh-food exposure in this cohort provided an opportunity to examine this association: 81% of respondents reported feeding at least some fresh food, 47% fed multiple diet types, and 12% reported that a given diet category comprised ≤10% of caloric intake. Such resolution would have been lost if diets had been reduced to a primary category or treated as binary exposures. At the same time, the recruitment source limits the generalizability of this finding. The cohort was enriched for consumers of a particular commercial fresh-food producer, although it included both current and prospective customers. Product-level information was insufficient to attribute the observed association to any specific brand or formulation. The finding should therefore be interpreted as pertaining to the broad fresh-food category as represented in this cohort and may not generalize to other commercial fresh-food populations. It should also not be extrapolated to home-prepared cooked diets, which constitute a different category of exposure, as they differ in formulation and quality-control practices and may be nutritionally inadequate if not appropriately formulated [57].

We also identified significant associations with both dry and wet foods. The positive association between dry food and overweight status accords with the existing literature [13,14,20,28,29]. One possible explanation for the dry food association is its higher energy density; on an as-fed basis, dry food contains approximately four times as many calories as wet food [58]. Consequently, even small amounts of overfeeding due to inaccurate portioning [59] can result in a substantial excess of caloric intake relative to energy requirements. Wet food, for which the literature is inconclusive [1,3,14,15,20,29], was associated with lower body condition category in the primary analysis, appearing both as a negative correlate of overweight and a positive correlate of underweight. Wet diets are less energy dense, which may increase the risk of inadvertent underfeeding if owners portion food by container or volume rather than by caloric content [60,61]. Aged cats fed a wet diet ad libitum consumed fewer calories than cats fed dry or mixed dry/wet diets, with caloric intake from wet diets falling below maintenance energy requirements, and intake from dry diets exceeding them [62]. However, wet diets may also be introduced in response to poor appetite, illness, or weight loss, raising the possibility of reverse causality [63]. The positive association of dry food with overweight and the negative association of wet food with overweight persisted when BCS 6 was reclassified as ideal, under a multinomial specification with ideal body condition as the reference, and after excluding kittens. Although the wet-food association with underweight was no longer statistically significant, the estimate remained directionally consistent and the fresh-food proportion was instead significantly associated with lower odds of underweight. Because these dietary proportions are interdependent and correlated, significance may shift between individual coefficients depending on how the shared compositional variation is partitioned in the model.

The consistency of the principal dietary associations was also supported by the stringent variable-stability criterion used in the primary analysis, under which predictors were required to be retained in 100% of bootstrap resamples within each imputed dataset. Together with their directional persistence across alternative BCS classification, multinomial modeling, and exclusion of kittens, this reduces, but does not eliminate, concern that the associations with overweight and ideal body condition are artifacts of a particular outcome specification or unstable variable selection, while the less stable underweight findings warrant more cautious interpretation. Because different sets of diet-format variables were retained across models, the dietary adjustment and implicit comparator composition also differed, potentially contributing to changes in individual associations. Individual dietary coefficients should therefore not be interpreted as direct pairwise comparisons between food formats or compared quantitatively across models as though they shared a common reference diet. In addition, as dietary formats are broad exposure categories rather than nutritionally uniform diets, individual dietary nutrient profiles were not characterized. Therefore, the analysis cannot distinguish associations attributable to macronutrient composition, moisture content, or energy density from those attributable to processing or other correlated product and feeding characteristics. These characteristics are possible explanations for the observed associations, not mechanistic pathways established by this study. The findings therefore should not be interpreted as effects of dietary format independent of nutritional composition or assumed to apply uniformly to all products within a format.

A key concern for our diet-type associations is that owners feeding different diets might systematically perceive or report their cats’ body condition differently, biasing the results. Previous work has shown that owner perception of BCS may be imperfect. Non-differential misclassification of BCS would be expected to attenuate associations and is therefore unlikely to explain observed differences. The more important concern is differential misclassification, whereby perception of body condition varies systematically across groups. To address this, we performed two robustness checks that exploit the two owner-reported adiposity measures collected: (1) BCS-derived weight status, based on owner assignment using pictorial and verbal descriptors, and (2) unguided owner perception. Although both are owner reported, BCS is the more standardized measure because it is anchored to visual and descriptive criteria, whereas unguided owner perception reflects an unstructured subjective judgment and may therefore be more susceptible to perception bias. If systematic perception bias were present, it would be expected to influence the more subjective unguided classification more strongly than the descriptor-based BCS, manifesting as differences in the relationship between the two measures across groups. First, we assessed whether owners who feed fresh diets might systematically misperceive or misreport their cats’ body condition as a consequence of greater financial and practical investment. We found no evidence that the relationship between BCS-derived status and unguided owner perception varied by food type across overweight, underweight, or ideal categories. Second, we examined whether owners feeding fresh diets were more likely to report engaging in weight-control practices, potentially reflecting unmeasured management behaviors. After adjusting for BCS-derived body condition and age group, there was again no evidence of differences in reported weight-control behavior across food types. Together, these analyses reduce concern that the observed diet associations are primarily driven by systematic perception bias or broad differences in owner engagement. However, they do not rule out more subtle variation in the intensity, quality, or effectiveness of weight-management practices not captured by the available measures.

We identified a negative association of free feeding with overweight and a positive association with ideal weight [15]. This result should be interpreted cautiously since owners may discontinue free feeding once their cats begin to gain weight, making current feeding style a consequence rather than a cause of body condition. It is also possible that the effect of ad libitum access differs by diet format, especially in a sample with a high prevalence of higher-moisture diets. Higher amounts of water consumption were associated with underweight which could be congruent with the observation that increased water content, typically as dietary moisture, ameliorates weight gain [64,65]. Greater water consumption may also be a marker of underlying disease processes, such as chronic kidney disease, diabetes mellitus, or hyperthyroidism, which are also associated with weight loss [63,66,67]. A secondary explanation is that increased water intake may promote gastric distension, increased activity, and transient satiety [65,68], potentially reducing food intake.

Additional sources of calories were not equivalent in their associations; asking more detailed questions may help reconcile conflicts in the literature [3,13,14,15,28,29]. Receiving extra cat food, being fed by other individuals, and receiving table scraps were associated with higher body condition, which is consistent with the idea that unmeasured or poorly accounted-for caloric intake contributes to overweight status. By contrast, hunting was associated with ideal body weight, potentially reflecting greater physical activity or food-seeking behavior in cats receiving less intentional feeding. Crunchy and freeze-dried treats were associated with ideal weight, whereas soft pâté-style treats were associated with underweight. However, the observed associations should be interpreted cautiously. One plausible explanation is that treat choice reflects the cat’s underlying health and feeding capacity rather than exerting a direct causal effect on weight; for example, cats with dental disease may be more likely to receive softer foods and less likely to consume harder treats. The same logic may apply to the association between probiotic or vitamin supplementation and lower body condition category, which may reflect treatment of pre-existing illness rather than a causal effect of the supplements themselves. Finally, sources of additional caloric intake were owner-reported and not independently verified. Discretionary feeding practices such as treats and table scraps may be incompletely reported because of recall or social-desirability bias, which could influence the observed associations.

Environmental factors were also associated with body condition. We recapitulate findings that certain types of outdoor access are negatively correlated with overweight [5,13,14,17,28] but also complicate the picture by noting that high percent of time spent outdoors is also correlated with being underweight, potentially through greater activity, more variable food intake, less owner oversight of feeding, or concurrent disease or parasite exposure. For this reason, cats spending extensive time outdoors may warrant closer monitoring of body condition and caloric intake. We also found that the type of outdoor access mattered: cats whose maximum access was limited to a porch or yard were more likely to be overweight than cats with no outdoor access. One possible explanation is that these environments provide less actual activity than owners expect, leading owners to overestimate energy expenditure and thus overfeed. However, as permitted access does not indicate how actively these spaces are used, the observed associations cannot be attributed specifically to differences in physical activity.

The indoor social and physical environment was likewise associated with body condition. The presence of other cats and dogs was significantly associated with lower body condition in both overweight and underweight models, supporting prior evidence [4,16]. This pattern is consistent with the possibility that cohabiting animals may increase play, stimulation, or competition, but it could also reflect stress, social hierarchy, or unequal access to food. A similar theme emerged with environmental enrichment: while not significant in the final models, all enrichment options considered were associated with ideal weight in the univariable analysis, though these relationships may reflect proxies for owner attentiveness. Overall, these findings are consistent with the broader hypothesis that both under- and overconditioning may arise in part from environmental dysregulation, whether through inactivity, boredom, stress, or disrupted feeding behavior.

Demographics-only models yielded associations broadly aligned with the prior literature, complementing the full models in which feeding and environmental variables were the primary exposures and demographic variables were retained as forced covariates. Age was strongly associated with body condition, consistent with prior studies. Neutering was not significantly associated with being overweight in the demographics-only model, the full model, or the multinomial model, whereas neutering was inversely associated with underweight relative to ideal body condition, consistent with previous findings [5]. However, neutering was significant in the univariable analysis, suggesting that variables retained in the model may capture more proximal behavioral pathways through which neutering influences weight, such as increased food-seeking, greater caloric intake, or reduced activity [69]. We also observed an interaction between sex and neuter status, with neutering associated with lower odds of underweight and higher odds of ideal BCS in females, while this association was attenuated in males, who were already less likely to be underweight when intact. This may be consistent with sex-specific endocrine responses to neutering [70]. Among female cats, a single litter is associated with overweight status, but not additional litters. This may be due to multiple litters arising from cats in breeding programs experiencing structured feeding practices and environment, or selection bias for cats with leaner, healthier phenotypes.

Breed-related findings similarly agree with prior work. Bengals and Ragdolls were more likely to be at a lower body condition, while British Shorthairs were more likely to be at a higher weight status, consistent with previous reports that British Shorthairs may be less active [43]. By grouping breeds according to origin, we also observed that cats in the Mediterranean group were more likely to be overweight, whereas those in the Asian group were more likely to be underweight. This pattern is biologically plausible insofar as breeds with shared origin may share a genetic background, and weight has a genetic component [71]. At the same time, breed-origin groupings may also proxy owner preferences or other unmeasured features, so the result may not be purely genetic. Results in mixed cats stratified by craniofacial morphology refine previous work identifying mixed-breed cats broadly as more likely to be overweight [3,8,9,14]. Dolichocephalic mixed cats tended toward lower body condition, mesocephalic mixed cats tended toward higher weight status, and brachycephalic mixed cats were significantly less likely to be at an ideal weight with non-significant bidirectional contributions. This discrepancy may reflect differences in breed composition across studies, or a more direct effect of morphology on body condition. For instance, brachycephalic cats are more prone to dental disorders [72] and exhibit more compact olfactory bulbs [73] which could affect food consumption. Additionally, preferences for certain craniofacial traits may be associated with differences in owner management; for example, individuals in animal care professions, who typically have greater experience with weight management, tend to show lower preference for brachycephalic cats [74].

Several limitations should be considered when interpreting these results. Foremost is the study population. Participants were recruited from customers of a direct-to-consumer fresh cat food company and likely differ from cat owners generally in socioeconomic characteristics, discretionary income, engagement with their cats’ nutrition, and attentiveness to feline health, which could affect both prevalence estimates and exposure–outcome associations. Relatedly, some cats may have been placed on a fresh diet in response to pre-existing health problems; while 64% of owners reported no diagnosed medical conditions, these reports were not independently verified, so the absence of a diagnosis does not imply the absence of disease. Prevalence estimates, though roughly aligned with prior findings [3,4,5,19], should not be extrapolated to the general cat population, as both are plausibly shifted by the recruitment mechanism and by participation bias. The dietary associations in particular should be read with the recruitment source in mind, although the sample did contain substantial variation in feeding practice, with 47% of cats fed multiple diet types and a wide distribution of caloric contributions across formats. Robustness analyses also found no evidence that associations by diet type were explained by systematic differences in owner weight perception or reported weight-control behavior, although these analyses cannot exclude residual confounding, reverse causation, or selection effects related to recruitment.

Second, both body-condition measures were owner-reported. The structured BCS item provided standardized pictorial and descriptive anchors but was not validated against veterinarian-assigned BCS in this study; therefore, differences between the two measures should be interpreted as classification discordance rather than diagnostic accuracy. Third, BCS captures adiposity rather than lean mass, and so may not fully characterize body composition in cats that are thin owing to muscle loss rather than low fat stores. Future studies should combine body-condition assessment with standardized muscle-condition scoring performed by trained assessors. Fourth, we did not collect certain owner-level variables previously linked to feline weight, and unmeasured owner behavior may contribute to the associations observed. Fifth, although the majority of the sample was not reported by their owners to have a diagnosed medical condition, underlying illness may influence body weight, activity, or appetite. We chose not to include health conditions in the primary models, for three reasons. Some conditions, such as diabetes or mobility limitations, may lie on the causal pathway from overweight to poorer health, so adjusting for them risks removing part of the association of interest. Modeling conditions individually is impractical, as many are sparse, correlated, and potentially unevenly diagnosed, while a single “any health issue” indicator would collapse biologically distinct conditions into a broad marker that may largely reflect age, overall illness burden, or frequency of veterinary contact. We likewise avoided excluding cats with health conditions, as this could introduce selection bias by removing clinically important overweight and underweight cats and would limit generalizability. Sixth, the large number of candidate associations examined and the use of data-dependent variable selection introduce additional uncertainty beyond that reflected in conventional confidence intervals and *p*-values. The reported inferential statistics are therefore model-conditional and nominal; they do not provide formal control of the false-positive rate arising from the broader screening and selection process. Accordingly, the analyses should be considered exploratory, and individual associations, particularly those with limited statistical support, should be regarded as hypothesis-generating and require confirmation in independent datasets. Finally, as in any cross-sectional study, reverse causality cannot be excluded. This is especially relevant for diet type, supplement use, and feeding practices, all of which may change in response to weight or illness. The findings do not establish that changing diet format or any other reported practice would cause a change in body condition, nor do they establish the efficacy or superiority of any commercial product. Replication in independently recruited, veterinarian-assessed cohorts with longitudinal follow-up will be important for determining which of the observed associations represent actionable targets for prevention and management.

## 5. Conclusions

This study identifies a broad and potentially modifiable set of feeding, activity, and household factors associated with feline body condition, and suggests that owner perception of cats’ weight can be shifted. Owners using a structured, picture- and description-based scoring tool identified fewer cats as ideal weight than they did through unguided judgment alone, and this gap was considerably wider for underweight than for overweight cats.

Among dietary factors, a higher proportion of fresh (mildly cooked) food was modestly associated with ideal body condition, and dry food with higher body condition, consistent with prior observational findings. Wet food was associated with lower body condition in the primary analysis, but not after excluding kittens or under a multinomial specification. Because wet food may be introduced in response to poor appetite, weight loss, or illness, this association should not be read as causal. Specific additional calorie sources (food from neighbors, other cats’ food, table scraps, and cat food given as treats) were associated with overweight status, whereas other sources were not. Because this was an observational study drawn from a self-selected population of fresh-food customers, these findings should be interpreted as hypothesis-generating rather than causal, and prospective studies in more representative populations are needed to confirm these associations and evaluate their clinical utility.

## Figures and Tables

**Table 1:** Distribution of owner-assessed body condition scores among 5552 cats using a 9-point body condition score scale. Scores of 1–3 were classified as underweight, scores of 4–5 as ideal weight, and scores of 6–9 as overweight or obese. Weight control refers to the percent of owners who report attempts to control their cats’ weight.

BCS Assessment	Unguided: Ideal		Unguided: Overweight		Unguided: Underweight		Unguided: Unsure	
	Count (%)	Weight Control (%)	Count (%)	Weight Control (%)	Count (%)	Weight Control (%)	Count (%)	Weight Control (%)
Ideal	2266 (77%)	895 (40%)	101 (3%)	90 (89%)	206 (7%)	160 (78%)	361 (12%)	216 (60%)
Overweight	548 (36%)	293 (54%)	647 (43%)	588 (91%)	16 (1%)	11 (69%)	297 (20%)	214 (72%)
Underweight	619 (56%)	174 (28%)	8 (1%)	5 (63%)	330 (30%)	235 (71%)	153 (14%)	56 (37%)

**Table 2:** Demographic associations with body condition (*n* = 5653). Multivariable logistic regression model examining associations between demographic factors and overweight, underweight, and ideal body condition, adjusted only for breed, age group, sex, neuter status, and their interaction, with no adjustment for feeding, activity, or environmental variables. Reference categories are Domestic Shorthair (breed), young adult (age group), female (sex), and not neutered. These estimates represent total demographic associations less attenuated by mediation through feeding and activity pathways than the primary models. *p*-values and 95% CIs are nominal and unadjusted for multiple comparisons.

		Overweight (*n* = 1542) vs. Not Overweight (*n* = 4111)		Underweight (*n* = 1134) vs. Not Underweight (*n* = 4519)		Ideal (*n* = 2977) vs. Not Ideal (*n* = 2676)	
		OR [95% CI]	*p*	OR [95% CI]	*p*	OR [95% CI]	*p*
Age group	Young Adult	1.00 [Reference]		1.00 [Reference]		1.00 [Reference]	
	Kitten	0.32 [0.18–0.58]	<0.001	1.68 [1.26–2.23]	<0.001	0.90 [0.69–1.16]	>0.05
	Mature Adult	2.12 [1.72–2.61]	<0.001	0.88 [0.70–1.10]	>0.05	0.66 [0.55–0.78]	<0.001
	Senior	1.69 [1.40–2.04]	<0.001	1.23 [1.03–1.47]	0.026	0.64 [0.55–0.74]	<0.001
	Geriatric	0.84 [0.64–1.08]	>0.05	2.71 [2.24–3.28]	<0.001	0.50 [0.42–0.59]	<0.001
Breed	Domestic Shorthair	1.00 [Reference]		1.00 [Reference]		1.00 [Reference]	
	Asian	0.32 [0.17–0.62]	<0.001	2.65 [1.94–3.62]	<0.001	0.65 [0.48–0.87]	0.004
	Bengal	0.43 [0.24–0.79]	0.006	2.15 [1.55–2.98]	<0.001	0.74 [0.54–1.01]	>0.05
	British Shorthair	1.83 [1.39–2.41]	<0.001	0.64 [0.47–0.88]	0.006	0.89 [0.71–1.12]	>0.05
	Domestic Long Hair	1.34 [0.96–1.86]	>0.05	0.86 [0.62–1.19]	>0.05	0.93 [0.72–1.21]	>0.05
	Domestic Medium Hair	1.30 [0.98–1.74]	>0.05	0.67 [0.49–0.91]	0.01	1.10 [0.87–1.38]	>0.05
	Hybrid	3.56 [1.14–11.12]	0.029	0.88 [0.19–4.01]	>0.05	0.41 [0.13–1.22]	>0.05
	Maine Coon	0.63 [0.33–1.19]	>0.05	0.95 [0.61–1.48]	>0.05	1.25 [0.85–1.83]	>0.05
	Mediterranean	4.42 [1.20–16.31]	0.026	0.55 [0.07–4.36]	>0.05	0.44 [0.13–1.55]	>0.05
	Mixed—Brachycephalic	2.64 [0.90–7.71]	>0.05	1.69 [0.58–4.91]	>0.05	0.35 [0.13–0.93]	0.036
	Mixed—Dolichocephalic	0.28 [0.13–0.62]	0.001	1.62 [1.13–2.32]	0.008	1.01 [0.72–1.41]	>0.05
	Mixed—Mesocephalic	1.32 [1.07–1.63]	0.009	0.81 [0.66–1.00]	0.05	0.98 [0.83–1.15]	>0.05
	Other	1.52 [1.12–2.07]	0.007	1.07 [0.80–1.44]	>0.05	0.75 [0.59–0.95]	0.019
	Ragdoll	0.49 [0.28–0.86]	0.013	1.04 [0.72–1.49]	>0.05	1.29 [0.93–1.77]	>0.05
	Western	0.94 [0.64–1.38]	>0.05	1.13 [0.82–1.56]	>0.05	0.95 [0.72–1.24]	>0.05
Sex	Male	0.97 [0.27–3.50]	>0.05	0.47 [0.28–0.81]	0.007	1.95 [1.16–3.26]	0.011
Neutering	Yes	2.39 [0.95–6.03]	>0.05	0.42 [0.28–0.63]	<0.001	1.71 [1.17–2.52]	0.006
Sex × Neutering	Male × Neutered	1.17 [0.32–4.25]	>0.05	1.88 [1.08–3.27]	0.027	0.52 [0.31–0.88]	0.015

**Table 3:** Multivariable logistic regression model examining factors associated with overweight body condition in cats (*n* = 5653). Odds ratios (ORs), 95% confidence intervals (CIs), and *p*-values are presented for retained variables in the final overweight versus non-overweight model. Reference categories are indicated as “Ref”. ORs > 1 indicate increased odds of being overweight, whereas ORs < 1 indicate reduced odds of being overweight relative to the reference category. *p*-values and 95% CIs are nominal and unadjusted for multiple comparisons; because predictors were retained through data-driven selection, estimates are conditional on the selected model and should be interpreted as exploratory. Age group, breed, sex, neuter status, and the sex–neuter interaction were forced a priori as adjustment variables and should not be interpreted as total demographic associations.

Variable		OR [CI]	*p*
Age group	Young adult	Ref	
	Kitten	0.21 [0.13–0.35]	<0.001
	Mature adult	1.73 [1.44–2.09]	<0.001
	Senior	1.38 [1.18–1.63]	<0.001
	Geriatric	0.72 [0.58–0.90]	0.003
Breed	Domestic Shorthair	Ref	
	Asian group	0.77 [0.48–1.24]	0.282
	Bengal	0.56 [0.35–0.91]	0.018
	British Shorthair	1.73 [1.36–2.21]	<0.001
	Domestic Long Hair	1.27 [0.95–1.69]	0.101
	Domestic Medium Hair	1.35 [1.05–1.74]	0.018
	Hybrid group	3.15 [0.93–10.74]	0.066
	Maine Coon	0.66 [0.40–1.08]	0.097
	Mediterranean group	5.02 [1.33–18.99]	0.018
	Mixed	1.24 [1.04–1.48]	0.014
	Other purebred	1.28 [0.97–1.70]	0.081
	Ragdoll	0.45 [0.29–0.70]	<0.001
	Western group	0.95 [0.69–1.31]	0.755
Sex	Female	Ref	
	Male	0.87 [0.33–2.30]	0.782
Neutered	Not neutered	Ref	
	Neutered	1.84 [0.93–3.64]	0.079
Sex × Neutered	Interaction	1.48 [0.56–3.93]	0.429
Feeding	Owner-estimated proportion of meal calories from dry food	1.83 [1.33–2.51]	<0.001
	Owner-estimated proportion of meal calories from wet food	0.67 [0.50–0.89]	0.005
	Free feeding	0.63 [0.52–0.76]	<0.001
	Fed additional calories from other household members	1.22 [0.98–1.53]	0.081
	Fed additional calories from neighbors	1.44 [1.12–1.84]	0.004
	Eats other cats’ food	1.50 [1.21–1.86]	<0.001
	Steals food	1.26 [0.98–1.61]	0.068
	Fed table scraps	1.31 [1.07–1.62]	0.010
	Receives additional cat food as a treat	1.49 [1.05–2.12]	0.025
	Given probiotics or prebiotics	0.78 [0.59–1.01]	0.062
Face shape	Mesocephalic	Ref	
	Brachycephalic	1.10 [0.69–1.77]	0.688
	Dolichocephalic	0.42 [0.33–0.53]	<0.001
Litter number	None (or male)	Ref	
	One	1.49 [1.07–2.05]	0.017
	Two or More	1.40 [0.89–2.21]	0.144
	Unsure	1.09 [0.82–1.45]	0.556
Maximum outdoor space access	None	Ref	
	Porch	1.35 [1.02–1.79]	0.038
	Yard	1.34 [1.04–1.73]	0.023
	Free-roaming	0.99 [0.77–1.28]	0.953
	Other	0.98 [0.71–1.36]	0.897
Cat’s pickiness about food	Not picky	Ref	
	Slightly picky	0.85 [0.72–1.00]	0.048
	Very picky	0.71 [0.59–0.86]	<0.001
Daily play frequency	Once a day or less	Ref	
	Twice a day	0.76 [0.64–0.92]	0.004
	Three times a day	0.71 [0.56–0.90]	0.005
	Four times a day or more	0.79 [0.65–0.96]	0.019
Time outdoors	None	Ref	
	1–25%	0.89 [0.71–1.12]	0.324
	25–50%	0.79 [0.61–1.02]	0.071
	50–75%	0.61 [0.45–0.82]	0.001
	75% or more	0.60 [0.39–0.90]	0.015
Household	Number of cats in household	0.88 [0.80–0.98]	0.015
	Number of dogs in household	0.82 [0.71–0.94]	0.006

**Table 4:** Multivariable logistic regression model examining factors associated with underweight body condition in cats (*n* = 5653). Odds ratios (ORs), 95% confidence intervals (CIs), and *p*-values are presented for retained variables in the final underweight versus non-underweight model. Reference categories are indicated as “Ref”. ORs > 1 indicate increased odds of being underweight, whereas ORs < 1 indicate reduced odds of being underweight relative to the reference category. *p*-values and 95% CIs are nominal and unadjusted for multiple comparisons; because predictors were retained through data-driven selection, estimates are conditional on the selected model and should be interpreted as exploratory. Age group, breed, sex, neuter status, and the sex–neuter interaction were forced a priori as adjustment variables and should not be interpreted as total demographic associations.

Variable		OR [CI]	*p*
Age group	Young adult	Ref	
	Kitten	1.78 [1.31–2.41]	<0.001
	Mature adult	0.90 [0.71–1.15]	0.409
	Senior	1.22 [1.00–1.49]	0.056
	Geriatric	2.61 [2.05–3.33]	<0.001
Breed	Domestic Shorthair	Ref	
	Asian group	1.46 [1.01–2.12]	0.044
	Bengal	1.42 [0.99–2.03]	0.057
	British Shorthair	0.68 [0.49–0.94]	0.020
	Domestic Long Hair	0.79 [0.56–1.11]	0.179
	Domestic Medium Hair	0.66 [0.48–0.91]	0.012
	Hybrid group	0.50 [0.10–2.45]	0.395
	Maine Coon	0.73 [0.45–1.18]	0.203
	Mediterranean group	0.49 [0.06–4.03]	0.509
	Mixed	0.82 [0.68–1.01]	0.057
	Other purebred	0.94 [0.69–1.28]	0.704
	Ragdoll	0.94 [0.64–1.38]	0.739
	Western group	0.90 [0.63–1.28]	0.546
Sex	Female	Ref	
	Male	0.43 [0.24–0.77]	0.005
Neutering	Not neutered	Ref	
	Neutered	0.47 [0.30–0.73]	<0.001
Sex ×Neutered	Interaction	1.97 [1.07–3.60]	0.028
Feeding	Owner-estimated proportion of meal calories from fresh food	0.90 [0.69–1.17]	0.435
	Owner-estimated proportion of meal calories from wet food	1.51 [1.06–2.16]	0.022
	Fed crunchy treats	0.88 [0.75–1.02]	0.092
	Fed freeze-dried treats	0.84 [0.71–1.00]	0.055
	Fed lickable treats	0.83 [0.69–1.00]	0.053
	Fed pate treats	2.14 [1.21–3.79]	0.009
	Fed prescription/therapeutic treats	0.57 [0.25–1.32]	0.189
	Fed additional calories from other household members	0.70 [0.52–0.96]	0.024
	Eats other cats’ food	0.73 [0.56–0.94]	0.015
	Fed table scraps	0.79 [0.61–1.03]	0.083
	Given probiotics or prebiotics	1.36 [1.04–1.78]	0.027
	Given vitamins	1.49 [1.07–2.09]	0.020
	Mesocephalic	Ref	
	Brachycephalic	1.78 [1.05–3.01]	0.032
Face shape	Dolichocephalic	2.04 [1.67–2.49]	<0.001
Meals per day	Not meal fed	Ref	
	One or two	0.88 [0.73–1.08]	0.220
	Three or more	0.77 [0.61–0.97]	0.028
Maximum outdoor space access	None	Ref	
	Porch	0.81 [0.58–1.14]	0.225
	Yard	0.86 [0.64–1.14]	0.295
	Free-roaming	0.98 [0.74–1.29]	0.883
	Other	1.15 [0.82–1.62]	0.413
Cat’s pickiness about food	Not picky	Ref	
	Slightly picky	1.56 [1.29–1.89]	<0.001
	Very picky	1.73 [1.40–2.13]	<0.001
Daily play frequency	Once a day or less		
	Twice a day	0.94 [0.76–1.17]	0.595
	Three times a day	1.15 [0.89–1.49]	0.293
	Four times a day or more	1.18 [0.95–1.46]	0.137
Daily sleep (hours)	<10	Ref	
	10–16	0.90 [0.74–1.10]	0.321
	16+	0.80 [0.63–1.01]	0.056
Time outdoors	None		
	1–25%	0.99 [0.77–1.28]	0.932
	25–50%	1.18 [0.89–1.57]	0.258
	50–75%	1.16 [0.84–1.61]	0.374
	75% or more	1.90 [1.26–2.86]	0.002
Observed water frequency	Less than daily	Ref	
	Daily	1.05 [0.87–1.27]	0.629
	More than daily	1.26 [1.04–1.52]	0.020
Household	Number of cats in household	1.20 [1.08–1.32]	<0.001
	Number of dogs in household	1.27 [1.12–1.44]	<0.001
	Scratching post/board access	0.88 [0.72–1.06]	0.177

**Table 5:** Multivariable logistic regression model examining factors associated with ideal body condition in cats (n = 5653). Odds ratios (ORs), 95% confidence intervals (CIs), and p-values are presented for retained variables in the final ideal versus non-ideal model. Reference categories are indicated as “Ref”. ORs > 1 indicate increased odds of being ideal, whereas ORs < 1 indicate reduced odds of being ideal relative to the reference category. p-values and 95% CIs are nominal and unadjusted for multiple comparisons; because predictors were retained through data-driven selection, estimates are conditional on the selected model and should be interpreted as exploratory. Age group, breed, sex, neuter status, and the sex–neuter interaction were forced a priori as adjustment variables and should not be interpreted as total demographic associations.

Variable		OR [CI]	*p*
Age group	Young adult	Ref	
	Kitten	1.33 [1.03–1.73]	0.031
	Mature adult	0.67 [0.56–0.79]	<0.001
	Senior	0.72 [0.62–0.83]	<0.001
	Geriatric	0.70 [0.58–0.84]	<0.001
Breed	Domestic Shorthair	Ref	
	Asian group	0.76 [0.55–1.05]	0.100
	Bengal	0.97 [0.71–1.33]	0.852
	British Shorthair	0.77 [0.62–0.97]	0.025
	Domestic Long Hair	0.98 [0.76–1.26]	0.857
	Domestic Medium Hair	0.99 [0.79–1.23]	0.906
	Hybrid group	0.60 [0.20–1.84]	0.373
	Maine Coon	1.40 [0.97–2.01]	0.073
	Mediterranean group	0.33 [0.08–1.32]	0.116
	Mixed	0.96 [0.82–1.11]	0.561
	Other purebred	0.89 [0.70–1.14]	0.367
	Ragdoll	1.53 [1.13–2.08]	0.006
	Western group	1.11 [0.84–1.45]	0.466
Sex	Female	Ref	
	Male	2.16 [1.29–3.63]	0.003
Neutering	Not neutered	Ref	
	Neutered	1.54 [1.04–2.29]	0.030
Sex × Neutered	Interaction	0.43 [0.25–0.73]	0.002
Feeding	Owner-estimated proportion of meal calories from fresh food	1.22 [1.04–1.42]	0.016
	Owner-estimated proportion of meal calories from raw food	2.19 [0.98–4.88]	0.056
	Receives additional cat food as a treat	0.72 [0.52–0.99]	0.043
	Fed crunchy treats	1.13 [1.01–1.26]	0.039
	Fed freeze-dried treats	1.20 [1.06–1.36]	0.004
	Fed pate treats	0.45 [0.27–0.76]	0.003
	Fed prescription/therapeutic treats	1.72 [0.97–3.06]	0.065
	Fed soft treats	1.15 [0.99–1.35]	0.076
	Free feeding	1.24 [1.07–1.43]	0.003
	Eats from garbage	1.54 [0.93–2.54]	0.091
	Hunts food	1.24 [1.07–1.44]	0.005
	Fed additional calories from neighbors	0.77 [0.62–0.96]	0.022
	Given vitamins	0.73 [0.55–0.96]	0.026
Face shape	Mesocephalic	Ref	
	Brachycephalic	0.65 [0.43–1.00]	0.052
	Dolichocephalic	1.04 [0.88–1.24]	0.618
Daily play frequency	Once a day or less	Ref	
	Twice a day	1.23 [1.05–1.43]	0.009
	Three times a day	1.17 [0.96–1.42]	0.115
	Four times a day or more	1.08 [0.92–1.28]	0.337
Stress frequency	None	Ref	
	Less than weekly	1.01 [0.87–1.18]	0.888
	Weekly or more	0.87 [0.73–1.04]	0.122
Observed water frequency	Less than daily	Ref	
	Daily	1.06 [0.93–1.22]	0.389
	More than daily	0.88 [0.77–1.02]	0.091
Household	Cat tower/climbing frame access	1.06 [0.94–1.19]	0.338

## Data Availability

The de-identified dataset and analysis code supporting this study have been deposited in Figshare. The records are available to editors and reviewers through a private peer-review link and will be made publicly available upon publication. Reserved DOIs: 10.6084/m9.figshare.33314820 and 10.6084/m9.figshare.33314862.

## Supplementary Materials

The following supporting information can be downloaded at: https://www.mdpi.com/article/10.3390/ani16213406/s1, Table S1: Questionnaire items and response options; Figure S1: Body condition score survey question presentation; Table S2: Breed groupings; Table S3: Outdoor access questions; Table S4: Variable missingness; Table S5: Cohort characteristics and univariable associations; Table S6: Frequency of reported medical conditions; Table S7: Variable inclusion frequency across bootstrapped samples; Table S8: Model diagnostics; Table S9: Robustness checks: owner perception and weight-control behavior; Table S10: Alternate overweight and ideal weight models (BCS 6 coded as ideal); Table S11: Alternate overweight, underweight, and ideal weight models (kittens excluded); Table S12: Multinomial regression results.
