# Supplementary material for: Diet Formats, Feeding Practices, and Environmental Factors Associated with Overweight, Underweight, and Ideal Body Condition in Household Cats
Source: Animals (Basel). 2026 Oct 28;16(21):3406. doi: 10.3390/ani16213406 (PMC13761520; doi:10.3390/ani16213406)
Supplement: Supplementary file 1 [file animals-16-03406-s001.zip › animals-4552567-supplementary.pdf]

Table S1: Questionnaire items and response options

Questionnaires were completed by participants while logged into their account portal. Items were presented sequentially across a series of webpages, with participants progressing through the questionnaires by clicking to continue to the next page. Questions were personalized using the cat's name and, where applicable, sex-specific wording, and some items were displayed conditionally based on responses to preceding questions.

Question	Answer Options	Notes
Which year was [cat name] born?	Year; Unsure.	
And which month?	Month; Unsure.	
This is an estimate.	True / False, separately for year and month.	
Are they male or female?	Female; Male.	
What was [cat name]'s last measured weight (in kg)?	Numeric entry, including decimals.	
When did [cat name] join your household?	Year; Unsure.	
And in which month?	Month; Unsure.	
This is an estimate.	True / False.	
How did [cat name] join your household?	Breeder; Private seller; Pet shop; Charity/rescue; Found; Born at home; Another household; Other.	
Has [cat name] been [spayed/neutered]?	Yes; No.	
At what age was [cat name] [spayed/neutered]?	Less than 7 weeks; 7–15 weeks; 16–23 weeks; 24 weeks or above; Unknown.	

Is [cat name] pregnant or lactating?	No; Yes, pregnant; Yes, lactating.	
How many litters has [cat name] had?	None; One; Two or more; Unknown.	
What breed is [cat name]?	Domestic short-hair; Domestic medium-hair; Domestic long-hair; British short-hair; British long-hair; Abyssinian; American Bobtail; American Curl; American shorthair; Balinese; Bengal; Birman; Burmese; Burmilla; Chausie; Chinchilla; Cornish Rex; Devon Rex; Exotic Short-hair; Japanese Bobtail; Korat; Kurilian Bobtail; LaPerm; Maine Coon; Manx; Moggie; Norwegian Forest Cat; Ocicat; Oriental Short-hair; Persian; Peterbald; Pixie Bob; Ragamuffin; Ragdoll; Russian Blue; Savannah; Scottish Fold; Siamese; Siberian Forest Cat; Singapura; Snowshoe; Somali; Sphynx; Tiffanie; Tonkinese; Turkish Angora; Turkish Van; Other; I don't know their breed.	
Is [cat name] any other breeds?	Yes; No; Unknown.	
And what breeds is [cat name] a mix of?	Multiple selection from the breed list above.	
How did you determine [cat name]'s breed?	Genetic test; Breeder; Shelter; Veterinarian; Appearance; Behaviour; Other.	
How do you know how old [cat name] is?	Had [cat name] since birth; Told by another party who has had [cat name] since birth; Shelter estimate; Veterinarian estimate; Other.	
What environment was [cat name] born in?	Current home; Prior parent's home (non-breeder); Professional breeder; Shelter; Other; Unknown.	
In what surroundings was [cat name] born?	Urban; Suburban; Rural; Unknown.	
What climate was [cat name] born in?	Tropical; Dry; Temperate; Continental; Polar; Unknown.	
What country was [he/she/they] born in?	Country dropdown; Unknown.	

What region? (If not sure, select 'Unsure'.)	Greater London; South East; South West; West Midlands; North West; North East; Yorkshire and the Humber; East Midlands; East of England; Unsure/Unknown.	Served if England.
What best describes their coat pattern?	Solid; Bicolour; Tortoiseshell; Calico; Colorpoint; Tabby; Shading; Other.	
What best describes [cat name]'s tabby coat pattern?	Classic; Spotted; Mackerel; Ticked (Agouti); Unsure.	Shown if Tabby selected.
What best describes [cat name]'s dominant coat colour?	White; Black; Orange/Red; Blue/Gray; Cream; Brown; Other.	
Does [cat name]'s coat have white spots?	No; Yes, mostly white; Yes, about half white, half another pattern; Yes, but mostly another pattern.	
What best describes [cat name]'s eye colour?	Yellow/amber; Hazel; Blue; Green; Other.	
What best describes [cat name]'s face shape?	Mesocephalic; Brachycephalic; Dolichocephalic.	
Has [cat name] been diagnosed by a vet with any of the following conditions?	Allergies; Arthritis; Asthma; Behavioural issues; Blindness; Cancer; Chronic diarrhoea; Chronic vomiting; Cystitis; Dental issues; Diabetes; Epilepsy; Feline Immunodeficiency Virus (FIV); Feline Leukaemia (FeLV); Inflammatory bowel disease; Heart disease; Hyperthyroidism; Kidney disease; Liver disease; Obesity; Pancreatitis; Urinary stones; Other; None.	
What is [cat name]'s main diet?	Wet (including canned); Dry; Raw; Fresh (including frozen); Other.	
About what portion of [cat name]'s regular meal calories are from [food type]?	A negligible amount (0–10%); Some (10–40%); About half (40–60%); Most (60–90%); Almost all (90–100%); Unsure.	Served for each diet type endorsed in main diet.

Are you serving [cat name] a therapeutic diet?	Yes; No.	
What therapeutic diet are you currently serving [cat name]?	Dental care; Weight management; Kidney care; Heart care; Urinary care; Joint care; Thyroid care; Liver care; Cancer care; Low-fat diet; Gastrointestinal care diet; Hydrolyzed diet; Novel protein diet; Limited/single intact protein diet; Behaviour management diet; Glucose management diet; Other.	
Is [selected therapeutic diet] a prescription diet?	Yes; No.	Served for every therapeutic diet endorsed.
When was the last time you changed your cat's diet?	Past week; Past month; Past year; Over a year ago; Never.	
How often do you make changes to [cat name]'s diet?	About monthly; A couple of times a year; Once a year or less.	
How many calories per day does [cat name] get from treats?	Numeric entry.	
And what treats does [cat name] get?	Soft treats; Crunchy treats; Freeze-dried treats; Jerky treats; Dental chews; Lickable treats; Pâté treats; Cat meal food given outside of regular mealtimes; Human food or table scraps; Bones; Prescription/therapeutic treats; Other.	
How often do you feed [selected treat type]?	Multiple times per day; Once daily; Several times per week; Weekly; Less than weekly; Never.	Served for every treat type endorsed.
Where else might [cat name] be getting calories?	Hunted food; Food given by neighbours; Garbage; Table scraps; Stolen food; Cat food eaten from other cats' portions; Food given by other household members without your knowledge; Other; Unsure; None of the above.	
Is [cat name]'s diet home-prepared?	Yes; Partially; No.	

While in your household, what has [cat name] been fed?	Wet (including canned); Dry; Raw; Fresh (including frozen); Other.	
In total, while in your care, about what portion of [cat name]'s cumulative calories have come from [food type]?	A negligible amount (0–10%); Some (10–40%); About half (40–60%); Most (60–90%); Almost all (90–100%); Unsure.	Served for every food type endorsed in household diet.
How fussy is [cat name]?	Eats all or almost all types of food tried; Eats some to most types of foods tried; Eats very few types of food tried.	
Typically, how long does [cat name] have access to their food?	Less than 5 minutes; 5–10 minutes; 10–30 minutes; 30–60 minutes; Several hours; I leave it out all day.	
How many meals does [cat name] receive per day?	One; Two; Three or more; I free feed.	
What supplements do you give [cat name]?	Probiotics or prebiotics; Vitamins; Fish oil; Other.	
What functional treats do you give [cat name]?	Dental treats; Hairball control treats; Joint treats; Urinary tract treats; Training treats; Gastrointestinal health treats; Treats to support weight loss; Behaviour modifying treats; Other functional treats; None of the above.	
Which, if any, cat food toppers or flavour enhancers do you add to [cat name]'s meals?	Liquid toppings (gravies, sauces or broths); Crunchy toppings (flakes, nibbles or dry shreds); Soft toppings (hydrated shreds, hydrated nibbles); Powders; Other; None.	
What proteins have you fed your cat in the past three months?	Chicken; Turkey; Duck; Salmon; Tuna; Mackerel; Whitefish; Shrimp; Pork; Beef; Lamb; Other.	
What wet food textures do you feed [cat name]?	Pâté; Chunks in gravy; Chunks in jelly; Chunks in broth; Shreds in gravy; Shreds in jelly; Shreds in broth; Flakes in jelly; Flakes in broth; Mousse; Cake.	
How do you feed [cat name]?	Meal feed; Timed feeder; Free feed; Other.	

And what do you feed [cat name] from?	Traditional food bowl; Toy/puzzle feed; Electronic feed; Hand feed; Other.	
How do you determine how much to feed [cat name]?	Customised meals; Package instructions; Veterinarian recommendation; Breeder recommendation; Shelter recommendation; Support group recommendation; Experience; Book; Course; Website: pet food; Website: veterinary practice; Website: pet blog; Other.	
How often do you see [cat name] drink water?	Never; Rarely; Occasionally; Daily; Several times a day; Constantly.	
What was [cat name]'s last measured weight (in kg)?	Numeric entry, including decimal.	
What do you believe is [cat name]'s ideal weight?	Numeric entry, including decimal.	
Do you consider [cat name] overweight?	Yes; No; I'm not sure.	
Do you consider [cat name] underweight?	Yes; No; I'm not sure.	
Have you struggled with [cat name] maintaining a healthy weight?	No; Yes, with a tendency to become overweight; Yes, with a tendency to become underweight.	
When did [cat name] become overweight?	Year; Unknown; Never.	Served if overweight
Has [cat name] previously been overweight?	Yes; No.	Served if neither overweight nor underweight.
When did [cat name] become underweight?	Year; Unknown; Never.	Served if underweight
Has [cat name] previously been underweight?	Yes; No.	
Have you made an effort to control [cat	Yes; No.	

name]'s weight?		
What tactics have you used to control [cat name]'s weight?	Feeding food specially formulated for weight loss; Changing feeding schedule; Changing food portions; Adjusting treats; Using food-dispensing toys; Encouraging exercise; Metabolic supplements; Satiety supplements; Medications; Other.	
How effective was [selected tactic]?	Very effective; Somewhat effective; Not effective.	Served for every tactic endorsed
What is your cat's body condition?	Nine-point body-condition scale, BCS 1–9, presented using visual and written descriptions.	
Has [cat name] been [spayed/neutered]?	Yes; No.	
At what age was [cat name] [spayed/neutered]?	Less than 7 weeks; 7–15 weeks; 16–23 weeks; 24 weeks or above; Not sure.	
Is [cat name] pregnant or lactating?	No; Yes, pregnant; Yes, lactating.	
How many litters has [cat name] had?	None; One; Two or more; Unknown.	
How much of their time does [cat name] spend outdoors?	None; 1–25%; 25–50%; 50–75%; 75% or more.	
What is the outdoor environment like?	Urban; Suburban; Rural.	
How many rooms of indoor space does [cat name] have access to?	1–10+ dropdown.	
What elevated areas does [cat name] have access to in the home?	Cat trees; Shelves and cabinets; Other.	

What type of access does your cat have to the outside environment?	Through a screened window or door; On a leash or in a stroller/backpack; Supervised free-roaming within a fenced-in yard; Unsupervised free-roaming within a fenced-in yard; Supervised free-roaming; Unsupervised free-roaming; Enclosed patio/catio, porch, or balcony; Open-air patio, porch, or balcony; Other.	
How much total time is your cat active on a given day?	Less than 5 minutes; 5–10 minutes; 10–15 minutes; 15–30 minutes; More than 30 minutes; Unsure.	
How often does your cat play with humans?	Rarely; Weekly; Every few days; Once a day; Twice a day; Three times a day; Four times or more; Unsure.	
How much total time does your cat spend playing with humans on an average day?	Less than 5 minutes; 5–10 minutes; 10–15 minutes; 15–30 minutes; More than 30 minutes; Unsure.	
What enrichment options does [cat name] have access to?	Cat bed; Scratching post/board; Cat tower/climbing frame; Tunnels/hideouts; Window seat/perch; Catnip (spray, toy, plant, dried); String/wand toy; Laser toy; Crinkly/squeaky toys; Interactive/puzzle toys; Electronic toy; Other; None.	
How many hours do you believe [cat name] spends sleeping per day, on average?	Only a few hours (<5 hrs); Some of the day (5–10 hrs); About half the day (10–16 hrs); Most of the day (16–20 hrs); Almost all day (20+ hrs).	
How often does [cat name] encounter stressors?	Multiple times a day; Once a day; Multiple times a week; Once a week; Rarely; Never.	
How many adults and young adults (13+ years) are there?	Numeric entry.	
How many young children (12 years and under) are there?	Numeric entry.	
Including [cat name], how many cats in total do you have?	Numeric entry.	

How many dogs do you have in total?	Numeric entry.

Figure S1: Body condition score survey question presentation

Body condition score question as presented in the survey. Descriptions are adapted from WSAVA and weight classifications (underweight, ideal, overweight) are removed to reduce bias.

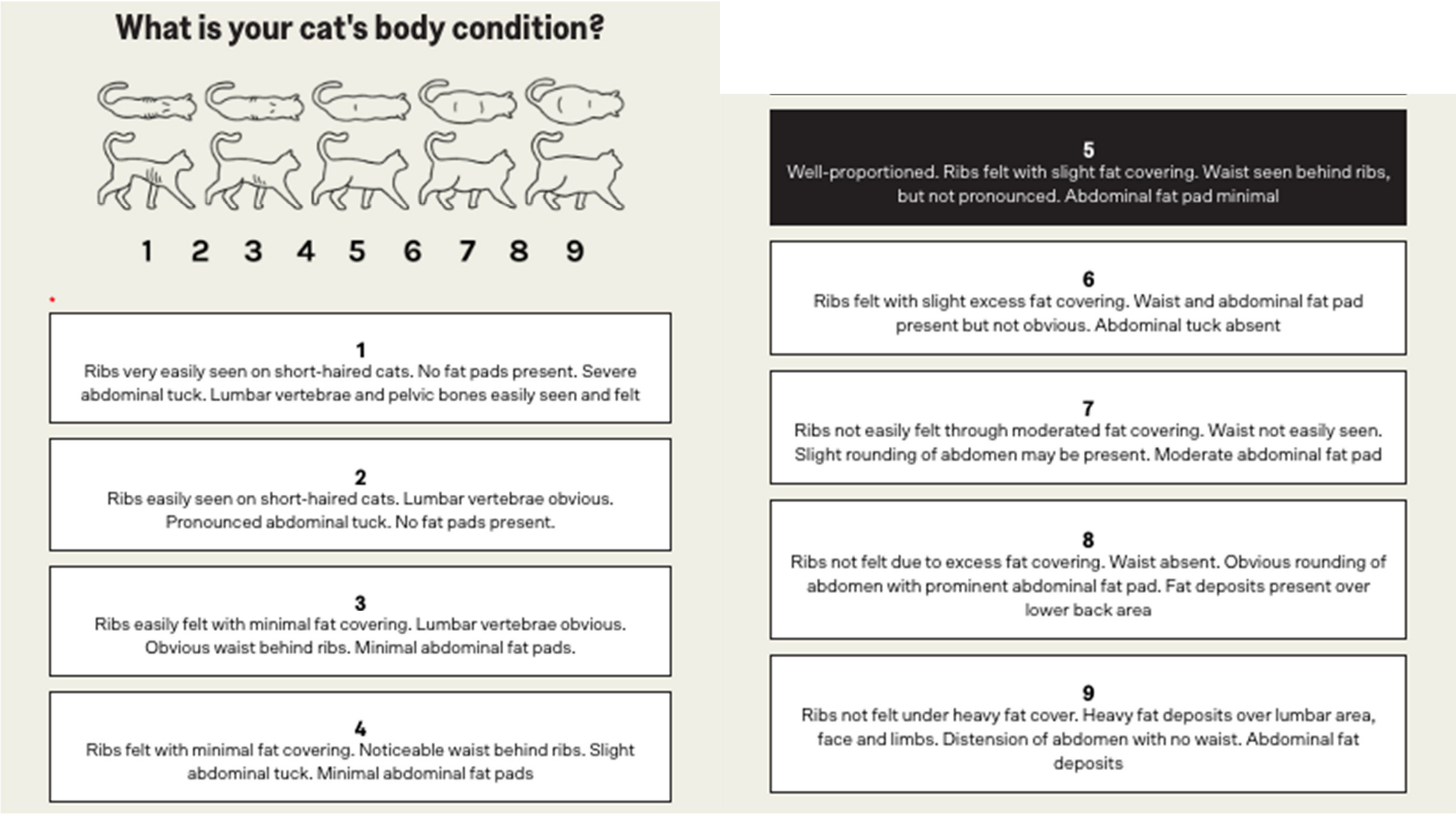

Table S2: Breed groupings

Reported breeds were grouped by shared ancestry

Western group	British Shorthair, Maine Coon, Norwegian Forest Cat, Persian, Russian Blue, Siberian Forest Cat, Sphynx, Ragdoll, Scottish Fold, American Bobtail, American Curl, British Longhair, Chinchilla, American Shorthair, Exotic Shorthair, Kurilian Bobtail, LaPerm, Manx, and Ragamuffin.
Mediterranean group	Turkish Van and Turkish Angora.
Asian group	Burmese, Siamese, Abyssinian, Devon Rex, Oriental Shorthair, Snowshoe, Tonkinese, Cornish Rex, Japanese Bobtail, Korat, Ocicat, Peterbald, Singapura, and Somali.
Hybrid group	Bengal, Savannah, Chausie, and Pixie-bob.

Table S3: Outdoor access questions

Original questions below were consolidated into two categorical variables as described in the main text.

How much of their time does [cat name] spend outdoors?	None;<br>1-25%;<br>25-50%;<br>50-75%;<br>75+ percent.
What type of access does your cat have to the outside environment?	Through a screened window or door;<br>On a leash or in a stroller/backpack;<br>Supervised free-roaming within a fenced-in yard;<br>Unsupervised free-roaming within a fenced-in yard;<br>Supervised free-roaming;<br>Unsupervised free-roaming;<br>Enclosed patio/catio, porch, or balcony;<br>Open-air patio, porch, or balcony;<br>Other.

Table S4: Variable missingness

Eighteen variables contained missing data arising from either selection of an “unknown” response option or incomplete survey responses.

Variable	N Missing	Percent Missing
Cats in Household	583	10.3%
Dogs in Household	583	10.3%
Number of Children in Household	583	10.3%
Number of Other Adults in Household	583	10.3%
Stress Frequency	573	10.3%
Sleep Duration	570	10.1%
Play Frequency	564	10.3%
Indoor Space	559	9.9%
Outdoor Environment Type	558	9.9%

Water Intake Frequency	554	9.8%
Pickiness	535	9.5%
Home-Cooked Food	372	6.6%
Daily Dry Food Calories	218	3.9%
Daily Fresh Food Calories	187	3.3%
Daily Wet Food Calories	145	2.6%
Daily Raw Food Calories	22	0.39%
Face Shape	20	0.35%
Daily Other Food Calories	19	0.34%

Table S5: Cohort characteristics and univariable associations

Cohort Characteristics (N=5653)								
		% of N (categorical) or mean (SD)	Overweight (N=1542) vs. Not Overweight (N=4111)		Underweight (N=1134) vs. Not Underweight (N=4519)		Ideal (N=2977) vs. Not Ideal (N=2676)	
			OR [95% CI]	p	OR [95% CI]	p	OR [95% CI]	p
Breed	Domestic Shorthair	37.0%	1.00 [Reference]	-	1.00 [Reference]	-	1.00 [Reference]	-
	Asian group	3.6%	0.41 [0.26–0.61]	<0.001	2.45 [1.81–3.32]	<0.001	0.86 [0.64–1.14]	>0.05
	Bengal	3.4%	0.34 [0.21–0.53]	<0.001	1.96 [1.42–2.69]	<0.001	1.13 [0.84–1.53]	>0.05
	British Shorthair	6.9%	1.69 [1.35–2.12]	<0.001	0.60 [0.44–0.82]	<0.01	0.83 [0.67–1.03]	>0.05
	Domestic Long Hair	5.0%	1.20 [0.91–1.57]	>0.05	0.90 [0.65–1.23]	>0.05	0.92 [0.71–1.18]	>0.05
	Domestic Medium Hair	6.9%	1.31 [1.04–1.65]	<0.05	0.68 [0.50–0.91]	<0.05	0.99 [0.80–1.23]	>0.05
	Hybrid group	0.2%	1.63 [0.49–4.92]	>0.05	0.72 [0.11–2.69]	>0.05	0.79 [0.25–2.37]	>0.05
	Maine Coon	2.7%	0.46 [0.29–0.71]	<0.001	0.89 [0.57–1.33]	>0.05	1.83 [1.30–2.61]	<0.001
	Mediterranean group	0.2%	3.92 [1.12–15.40]	<0.05	0.44 [0.02–2.35]	>0.05	0.39 [0.08–1.42]	>0.05
	Mixed	19.8%	1.06 [0.90–1.24]	>0.05	0.93 [0.77–1.11]	>0.05	1.00 [0.87–1.16]	>0.05
	Other purebred	5.7%	1.05 [0.81–1.36]	>0.05	1.06 [0.79–1.41]	>0.05	0.92 [0.73–1.17]	>0.05
	Ragdoll	3.9%	0.42 [0.28–0.61]	<0.001	0.97 [0.68–1.37]	>0.05	1.82 [1.36–2.46]	<0.001
	Western group	4.9%	0.81 [0.60–1.08]	>0.05	1.01 [0.73–1.37]	>0.05	1.18 [0.91–1.52]	>0.05
Age group	Young adult	42.0%	1.00 [Reference]	-	1.00 [Reference]	-	1.00 [Reference]	-
	Kitten	6.4%	0.17 [0.11–0.27]	<0.001	2.05 [1.59–2.64]	<0.001	1.36 [1.08–1.72]	<0.01
	Mature adult	13.8%	1.90 [1.60–2.25]	<0.001	0.83 [0.66–1.04]	>0.05	0.63 [0.54–0.74]	<0.001
	Senior	24.0%	1.48 [1.28–1.72]	<0.001	1.16 [0.97–1.37]	>0.05	0.66 [0.58–0.76]	<0.001
	Geriatric	13.9%	0.76 [0.62–0.92]	<0.01	2.52 [2.10–3.03]	<0.001	0.61 [0.52–0.72]	<0.001
Sex	Female	48.7%	1.00 [Reference]	-	1.00 [Reference]	-	1.00 [Reference]	-
	Male	51.3%	1.16 [1.03–1.30]	<0.05	0.83 [0.73–0.94]	<0.01	1.00 [0.90–1.11]	>0.05
Neutering	No	4.7%	1.00 [Reference]	-	1.00 [Reference]	-	1.00 [Reference]	-
	Yes	95.3%	4.38 [2.89–7.00]	<0.001	0.53 [0.41–0.69]	<0.001	0.72 [0.56–0.93]	<0.05
Free feeding	No	80.9%	1.00 [Reference]	-	1.00 [Reference]	-	1.00 [Reference]	-
	Yes	19.1%	0.60 [0.50–0.70]	<0.001	1.38 [1.18–1.61]	<0.001	1.18 [1.03–1.34]	<0.05
Time outdoors	None	35.2%	1.00 [Reference]	-	1.00 [Reference]	-	1.00 [Reference]	-
	1–25%	29.9%	1.17 [1.01–1.35]	<0.05	0.86 [0.73–1.02]	>0.05	0.97 [0.85–1.10]	>0.05
	25–50%	20.1%	1.06 [0.90–1.25]	>0.05	0.92 [0.76–1.10]	>0.05	1.01 [0.87–1.17]	>0.05
	50–75%	11.4%	0.82 [0.67–1.01]	>0.05	0.89 [0.71–1.11]	>0.05	1.25 [1.04–1.49]	<0.05
	75%+	3.3%	0.82 [0.57–1.15]	>0.05	1.44 [1.02–2.00]	<0.05	0.89 [0.66–1.20]	>0.05
Face shape	Mesocephalic	83.6%	1.00 [Reference]	-	1.00 [Reference]	-	1.00 [Reference]	-
	Brachycephalic	1.7%	1.10 [0.71–1.68]	>0.05	1.34 [0.81–2.13]	>0.05	0.76 [0.51–1.14]	>0.05
	Dolichocephalic	14.6%	0.36 [0.29–0.45]	<0.001	2.30 [1.95–2.71]	<0.001	1.04 [0.89–1.20]	>0.05
Birth environment	Professional breeder	18.6%	1.00 [Reference]	-	1.00 [Reference]	-	1.00 [Reference]	-
	Current home	3.2%	1.25 [0.86–1.79]	>0.05	1.11 [0.77–1.59]	>0.05	0.79 [0.58–1.08]	>0.05
	Other	8.5%	1.24 [0.96–1.60]	>0.05	0.93 [0.72–1.21]	>0.05	0.90 [0.73–1.12]	>0.05
	Other home	35.2%	1.44 [1.21–1.73]	<0.001	0.81 [0.68–0.97]	<0.05	0.88 [0.76–1.02]	>0.05
	Shelter	3.0%	1.41 [0.97–2.04]	>0.05	0.91 [0.61–1.34]	>0.05	0.83 [0.60–1.15]	>0.05
	Unknown	31.5%	1.82 [1.52–2.18]	<0.001	0.71 [0.58–0.85]	<0.001	0.79 [0.68–0.92]	<0.01
Birth surroundings	Suburban	27.6%	1.00 [Reference]	-	1.00 [Reference]	-	1.00 [Reference]	-

	Rural	15.0%	0.86 [0.71–1.05]	>0.05	1.16 [0.95–1.42]	>0.05	1.01 [0.85–1.19]	>0.05
	Unknown	33.8%	1.27 [1.10–1.48]	<0.01	0.93 [0.78–1.10]	>0.05	0.86 [0.75–0.98]	<0.05
	Urban	23.6%	0.98 [0.83–1.15]	>0.05	1.00 [0.83–1.20]	>0.05	1.02 [0.88–1.18]	>0.05
Litter history	None (or male)	88.8%	1.00 [Reference]	-	1.00 [Reference]	-	1.00 [Reference]	-
	One	3.8%	1.31 [0.97–1.74]	>0.05	0.95 [0.67–1.33]	>0.05	0.82 [0.63–1.08]	>0.05
	Two or more	2.0%	1.19 [0.78–1.77]	>0.05	0.96 [0.59–1.52]	>0.05	0.89 [0.61–1.29]	>0.05
	Unsure	5.4%	1.17 [0.90–1.50]	>0.05	0.87 [0.64–1.17]	>0.05	0.96 [0.76–1.21]	>0.05
Receives home-cooked food	No	78.8%	1.00 [Reference]	-	1.00 [Reference]	-	1.00 [Reference]	-
	Partially	10.5%	0.90 [0.73–1.10]	>0.05	0.98 [0.78–1.22]	>0.05	1.11 [0.93–1.32]	>0.05
	Yes	10.7%	1.11 [0.91–1.34]	>0.05	1.03 [0.82–1.28]	>0.05	0.91 [0.76–1.08]	>0.05
Play frequency	Once a day or less	53.9%	1.00 [Reference]	-	1.00 [Reference]	-	1.00 [Reference]	-
	Twice a day	19.1%	0.75 [0.63–0.88]	<0.001	0.87 [0.72–1.05]	>0.05	1.37 [1.18–1.59]	<0.001
	Three times a day	10.4%	0.62 [0.49–0.77]	<0.001	1.14 [0.90–1.43]	>0.05	1.34 [1.11–1.62]	<0.01
	Four times or more a day	16.5%	0.59 [0.49–0.70]	<0.001	1.31 [1.09–1.58]	<0.01	1.26 [1.08–1.47]	<0.01
Play duration	0–15 minutes	53.3%	1.00 [Reference]	-	1.00 [Reference]	-	1.00 [Reference]	-
	15–30 minutes	16.3%	0.86 [0.73–1.02]	>0.05	1.01 [0.83–1.22]	>0.05	1.12 [0.97–1.30]	>0.05
	30+ minutes	30.4%	0.71 [0.61–0.81]	<0.001	1.26 [1.09–1.46]	<0.01	1.13 [1.00–1.27]	>0.05
Meals per day	One or two	56.0%	1.00 [Reference]	-	1.00 [Reference]	-	1.00 [Reference]	-
	Three or more	22.5%	0.98 [0.85–1.13]	>0.05	1.04 [0.88–1.23]	>0.05	0.99 [0.87–1.13]	>0.05
	Not meal fed	21.5%	0.71 [0.61–0.83]	<0.001	1.54 [1.32–1.80]	<0.001	0.97 [0.85–1.10]	>0.05
Pickiness	Not picky	25.7%	1.00 [Reference]	-	1.00 [Reference]	-	1.00 [Reference]	-
	Slightly picky	47.7%	0.86 [0.74–0.99]	<0.05	1.46 [1.22–1.76]	<0.001	0.92 [0.80–1.05]	>0.05
	Very picky	26.7%	0.67 [0.56–0.79]	<0.001	1.89 [1.55–2.30]	<0.001	0.93 [0.80–1.08]	>0.05
Food delivery method	Traditional	81.3%	1.00 [Reference]	-	1.00 [Reference]	-	1.00 [Reference]	-
	Electronic	3.4%	0.88 [0.63–1.22]	>0.05	0.84 [0.57–1.21]	>0.05	1.22 [0.92–1.64]	>0.05
	Hand-fed	0.6%	1.20 [0.54–2.47]	>0.05	1.33 [0.56–2.85]	>0.05	0.71 [0.34–1.42]	>0.05
	Other	13.3%	0.91 [0.76–1.08]	>0.05	1.12 [0.93–1.35]	>0.05	1.00 [0.86–1.16]	>0.05
	Toy	1.4%	1.19 [0.72–1.91]	>0.05	0.40 [0.17–0.81]	<0.05	1.35 [0.85–2.15]	>0.05
Water frequency	Less than once daily	43.5%	1.00 [Reference]	-	1.00 [Reference]	-	1.00 [Reference]	-
	Once daily	28.3%	0.87 [0.75–1.01]	>0.05	1.11 [0.94–1.32]	>0.05	1.05 [0.92–1.20]	>0.05
	More than once daily	28.2%	0.84 [0.72–0.98]	<0.05	1.60 [1.36–1.88]	<0.001	0.84 [0.74–0.96]	<0.05
Outdoor environment	Suburban	51.1%	1.00 [Reference]	-	1.00 [Reference]	-	1.00 [Reference]	-
	Rural	22.4%	0.80 [0.68–0.94]	<0.01	1.18 [0.99–1.39]	>0.05	1.07 [0.93–1.23]	>0.05
	Urban	26.6%	0.96 [0.83–1.11]	>0.05	0.97 [0.82–1.15]	>0.05	1.05 [0.92–1.20]	>0.05
Stress frequency	Never	16.8%	1.00 [Reference]	-	1.00 [Reference]	-	1.00 [Reference]	-
	Less than weekly	51.9%	1.05 [0.88–1.26]	>0.05	1.02 [0.84–1.24]	>0.05	0.95 [0.81–1.11]	>0.05
	Weekly or more	31.4%	1.28 [1.06–1.55]	<0.05	0.98 [0.79–1.21]	>0.05	0.83 [0.70–0.98]	<0.05
Sleep amount	<10 hours	20.9%	1.00 [Reference]	-	1.00 [Reference]	-	1.00 [Reference]	-
	10–16 hours	47.6%	1.25 [1.06–1.48]	<0.01	0.85 [0.71–1.02]	>0.05	0.94 [0.81–1.08]	>0.05
	16+ hours	31.6%	1.38 [1.16–1.65]	<0.001	0.97 [0.80–1.18]	>0.05	0.79 [0.68–0.92]	<0.01
Receives crunchy treats	No	53.2%	1.00 [Reference]	-	1.00 [Reference]	-	1.00 [Reference]	-
	Yes	46.8%	1.04 [0.93–1.17]	>0.05	0.81 [0.71–0.93]	<0.01	1.10 [0.99–1.22]	>0.05
Receives freeze-dried treats	No	70.7%	1.00 [Reference]	-	1.00 [Reference]	-	1.00 [Reference]	-
	Yes	29.3%	0.90 [0.79–1.03]	>0.05	0.75 [0.64–0.86]	<0.001	1.30 [1.16–1.46]	<0.001
Receives lickable treats	No	80.4%	1.00 [Reference]	-	1.00 [Reference]	-	1.00 [Reference]	-
	Yes	19.6%	0.96 [0.83–1.11]	>0.05	0.85 [0.72–1.01]	>0.05	1.14 [1.00–1.30]	>0.05

Receives soft treats	No	85.7%	1.00 [Reference]	-	1.00 [Reference]	-	1.00 [Reference]	-
	Yes	14.3%	0.84 [0.71–1.00]	>0.05	1.01 [0.84–1.22]	>0.05	1.13 [0.98–1.32]	>0.05
Receives human food/table scraps as treats	No	90.8%	1.00 [Reference]	-	1.00 [Reference]	-	1.00 [Reference]	-
	Yes	9.2%	1.06 [0.86–1.29]	>0.05	1.01 [0.80–1.26]	>0.05	0.95 [0.79–1.14]	>0.05
Receives dental chews	No	92.6%	1.00 [Reference]	-	1.00 [Reference]	-	1.00 [Reference]	-
	Yes	7.4%	0.98 [0.78–1.22]	>0.05	0.87 [0.67–1.12]	>0.05	1.11 [0.91–1.36]	>0.05
Receives jerky treats	No	96.6%	1.00 [Reference]	-	1.00 [Reference]	-	1.00 [Reference]	-
	Yes	3.4%	1.08 [0.78–1.47]	>0.05	1.03 [0.71–1.45]	>0.05	0.92 [0.69–1.23]	>0.05
Receives cat meal food outside mealtimes as treats	No	96.9%	1.00 [Reference]	-	1.00 [Reference]	-	1.00 [Reference]	-
	Yes	3.1%	1.51 [1.09–2.06]	<0.05	1.01 [0.68–1.46]	>0.05	0.70 [0.51–0.94]	<0.05
Receives pate treats	No	98.8%	1.00 [Reference]	-	1.00 [Reference]	-	1.00 [Reference]	-
	Yes	1.2%	1.06 [0.60–1.77]	>0.05	2.11 [1.25–3.46]	<0.01	0.53 [0.32–0.87]	<0.05
Receives prescription/therapeutic treats	No	99.1%	1.00 [Reference]	-	1.00 [Reference]	-	1.00 [Reference]	-
	Yes	0.9%	1.08 [0.57–1.94]	>0.05	0.62 [0.25–1.29]	>0.05	1.23 [0.71–2.16]	>0.05
Eats from timed feeder	No	94.3%	1.00 [Reference]	-	1.00 [Reference]	-	1.00 [Reference]	-
	Yes	5.7%	1.41 [1.11–1.79]	<0.01	0.62 [0.44–0.84]	<0.01	0.98 [0.78–1.23]	>0.05
Eats from garbage	No	98.7%	1.00 [Reference]	-	1.00 [Reference]	-	1.00 [Reference]	-
	Yes	1.3%	1.12 [0.66–1.85]	>0.05	0.50 [0.22–0.99]	<0.05	1.31 [0.82–2.12]	<0.05
Fed additional calories from other household members	No	92.0%	1.00 [Reference]	-	1.00 [Reference]	-	1.00 [Reference]	-
	Yes	8.0%	1.50 [1.22–1.83]	<0.001	0.55 [0.41–0.73]	<0.001	0.98 [0.81–1.19]	>0.05
Hunts food	No	80.6%	1.00 [Reference]	-	1.00 [Reference]	-	1.00 [Reference]	-
	Yes	19.4%	0.94 [0.81–1.09]	>0.05	0.85 [0.72–1.01]	>0.05	1.16 [1.02–1.32]	<0.05
Fed additional calories from neighbours	No	92.9%	1.00 [Reference]	-	1.00 [Reference]	-	1.00 [Reference]	-
	Yes	7.1%	1.51 [1.22–1.86]	<0.001	0.76 [0.58–1.00]	>0.05	0.83 [0.68–1.01]	>0.05
Eats other cats' food	No	88.2%	1.00 [Reference]	-	1.00 [Reference]	-	1.00 [Reference]	-
	Yes	11.8%	1.34 [1.12–1.59]	<0.01	0.80 [0.64–0.98]	<0.05	0.90 [0.77–1.06]	>0.05
Steals food	No	93.2%	1.00 [Reference]	-	1.00 [Reference]	-	1.00 [Reference]	-
	Yes	6.8%	1.51 [1.21–1.88]	<0.001	0.67 [0.50–0.89]	<0.01	0.88 [0.72–1.08]	>0.05
Gets table scraps	No	90.3%	1.00 [Reference]	-	1.00 [Reference]	-	1.00 [Reference]	-
	Yes	9.7%	1.45 [1.20–1.74]	<0.001	0.74 [0.58–0.94]	<0.05	0.87 [0.73–1.04]	>0.05
Gets additional calories from other sources	No	94.7%	1.00 [Reference]	-	1.00 [Reference]	-	1.00 [Reference]	-
	Yes	5.3%	1.24 [0.96–1.59]	>0.05	0.88 [0.64–1.18]	>0.05	0.91 [0.72–1.14]	>0.05
Takes fish oil supplements	No	92.0%	1.00 [Reference]	-	1.00 [Reference]	-	1.00 [Reference]	-
	Yes	8.0%	0.96 [0.77–1.19]	>0.05	0.86 [0.67–1.10]	>0.05	1.13 [0.93–1.37]	>0.05
Takes probiotics or prebiotics	No	92.9%	1.00 [Reference]	-	1.00 [Reference]	-	1.00 [Reference]	-
	Yes	7.1%	0.78 [0.61–0.99]	<0.05	1.28 [1.00–1.62]	<0.05	1.02 [0.83–1.25]	>0.05
Takes vitamins	No	95.9%	1.00 [Reference]	-	1.00 [Reference]	-	1.00 [Reference]	-
	Yes	4.1%	0.94 [0.69–1.26]	>0.05	1.60 [1.19–2.14]	<0.01	0.75 [0.57–0.97]	<0.05
Cat tower/climbing frame	No	50.9%	1.00 [Reference]	-	1.00 [Reference]	-	1.00 [Reference]	-
	Yes	49.1%	0.84 [0.74–0.94]	<0.01	0.87 [0.77–0.99]	<0.05	1.26 [1.13–1.40]	<0.001
Scratching post	No	23.4%	1.00 [Reference]	-	1.00 [Reference]	-	1.00 [Reference]	-

	Yes	76.6%	0.99 [0.86–1.13]	>0.05	0.72 [0.63–0.84]	<0.001	1.26 [1.11–1.42]	<0.001
String/wand toy	No	34.3%	1.00 [Reference]	-	1.00 [Reference]	-	1.00 [Reference]	-
	Yes	65.7%	1.05 [0.93–1.19]	>0.05	0.71 [0.62–0.81]	<0.001	1.20 [1.08–1.34]	<0.01
Tunnels/hideouts	No	60.5%	1.00 [Reference]	-	1.00 [Reference]	-	1.00 [Reference]	-
	Yes	39.5%	0.85 [0.75–0.96]	<0.01	0.88 [0.77–1.00]	>0.05	1.24 [1.11–1.38]	<0.001
Window seat/perch	No	30.4%	1.00 [Reference]	-	1.00 [Reference]	-	1.00 [Reference]	-
	Yes	69.6%	0.96 [0.85–1.09]	>0.05	0.81 [0.71–0.93]	<0.01	1.18 [1.06–1.33]	<0.01
Maximum outdoor space access	None	29.0%	1.00 [Reference]	-	1.00 [Reference]	-	1.00 [Reference]	-
	Free-roaming	40.4%	1.03 [0.90–1.19]	>0.05	0.89 [0.77–1.04]	>0.05	1.05 [0.92–1.19]	>0.05
	Other	5.6%	0.80 [0.60–1.06]	>0.05	0.94 [0.70–1.26]	>0.05	1.23 [0.97–1.57]	>0.05
	Porch access	7.0%	1.17 [0.91–1.49]	>0.05	0.74 [0.55–0.98]	<0.05	1.07 [0.86–1.34]	>0.05
	Yard access	18.0%	1.26 [1.06–1.50]	<0.01	0.80 [0.66–0.97]	<0.05	0.95 [0.82–1.11]	>0.05
Access to outdoors unsupervised	No	54.3%	1.00 [Reference]	-	1.00 [Reference]	-	1.00 [Reference]	-
	Yes	45.7%	1.07 [0.95–1.20]	>0.05	0.97 [0.86–1.11]	>0.05	0.96 [0.87–1.07]	>0.05
% calories from dry	None	65.4%	1.00 [Reference]	-	1.00 [Reference]	-	1.00 [Reference]	-
	0–10%	8.7%	0.94 [0.75–1.17]	>0.05	1.07 [0.84–1.35]	>0.05	1.00 [0.83–1.21]	>0.05
	10–40%	14.0%	1.12 [0.94–1.33]	>0.05	0.92 [0.75–1.12]	>0.05	0.96 [0.82–1.13]	>0.05
	40–60%	8.8%	1.03 [0.83–1.28]	>0.05	0.86 [0.67–1.10]	>0.05	1.07 [0.88–1.30]	>0.05
	60–90%	2.1%	1.62 [1.10–2.37]	<0.05	0.87 [0.53–1.38]	>0.05	0.72 [0.49–1.04]	>0.05
	90–100%	0.9%	1.78 [0.99–3.11]	<0.05	1.08 [0.53–2.05]	>0.05	0.57 [0.32–0.99]	<0.05
% calories from fresh	None	19.2%	1.00 [Reference]	-	1.00 [Reference]	-	1.00 [Reference]	-
	0–10%	1.0%	1.04 [0.54–1.91]	>0.05	1.29 [0.68–2.36]	>0.05	0.79 [0.44–1.39]	>0.05
	10–40%	4.5%	0.97 [0.70–1.34]	>0.05	0.94 [0.67–1.30]	>0.05	1.07 [0.81–1.42]	>0.05
	40–60%	13.3%	0.96 [0.77–1.20]	>0.05	0.75 [0.59–0.95]	<0.05	1.25 [1.03–1.52]	<0.05
	60–90%	20.5%	1.03 [0.85–1.25]	>0.05	0.67 [0.54–0.83]	<0.001	1.27 [1.07–1.51]	<0.01
	90–100%	41.5%	1.08 [0.91–1.28]	>0.05	0.68 [0.57–0.82]	<0.001	1.21 [1.04–1.41]	<0.05
% calories from wet	None	78.0%	1.00 [Reference]	-	1.00 [Reference]	-	1.00 [Reference]	-
	0–10%	2.1%	0.81 [0.51–1.24]	>0.05	1.10 [0.68–1.72]	>0.05	1.11 [0.76–1.62]	>0.05
	10–40%	6.4%	0.86 [0.66–1.10]	>0.05	1.20 [0.92–1.56]	>0.05	1.00 [0.80–1.25]	>0.05
	40–60%	6.4%	1.06 [0.83–1.35]	>0.05	1.16 [0.88–1.50]	>0.05	0.87 [0.70–1.08]	>0.05
	60–90%	4.5%	0.68 [0.49–0.92]	<0.05	1.52 [1.13–2.03]	<0.01	0.99 [0.77–1.28]	>0.05
	90–100%	2.6%	0.60 [0.38–0.90]	<0.05	1.45 [0.97–2.11]	>0.05	1.12 [0.80–1.57]	>0.05
% calories from raw	None	97.5%	1.00 [Reference]	-	1.00 [Reference]	-	1.00 [Reference]	-
	0–10%	1.0%	0.52 [0.24–1.00]	>0.05	1.24 [0.64–2.25]	>0.05	1.36 [0.80–2.37]	>0.05
	10–40%	0.7%	1.42 [0.72–2.68]	>0.05	0.85 [0.34–1.81]	>0.05	0.82 [0.44–1.53]	>0.05
	40–60%	0.3%	0.96 [0.27–2.81]	>0.05	1.45 [0.40–4.26]	>0.05	0.79 [0.28–2.21]	>0.05
	60–90%	0.1%	0.00 [–-31.30]	>0.05	1.33 [0.20–5.79]	>0.05	2.72 [0.63–18.56]	>0.05
	90–100%	0.4%	0.28 [0.04–0.96]	<0.05	0.67 [0.16–1.97]	>0.05	2.90 [1.13–8.87]	<0.05
Cats in household	Numeric	1.41 [0.80]	0.93 [0.86–1.00]	>0.05	1.16 [1.07–1.26]	<0.001	0.96 [0.90–1.03]	>0.05
Dogs in household	Numeric	0.19 [0.53]	0.79 [0.69–0.89]	<0.001	1.38 [1.23–1.54]	<0.001	0.94 [0.85–1.04]	>0.05
Indoor space access (number of rooms)	Numeric	5.85 [2.00]	0.98 [0.95–1.01]	>0.05	1.00 [0.97–1.04]	>0.05	1.02 [0.99–1.04]	>0.05
Children in household	Numeric	0.20 [0.55]	0.88 [0.78–0.99]	<0.05	1.15 [1.02–1.29]	<0.05	1.01 [0.91–1.11]	>0.05
Adults in household	Numeric	1.97 [0.97]	1.00 [0.94–1.07]	>0.05	0.99 [0.92–1.06]	>0.05	1.00 [0.95–1.06]	>0.05

Table S6: Frequency of reported medical conditions

Disease Diagnosis	N Reported	Percent of Cohort (%)
None Reported	3,601	63.71
Dental Issues	639	11.21
Other Conditions	448	7.86
Allergies	298	5.23
Arthritis	288	5.05
Hyperthyroidism	220	3.86
Kidney Disease	125	2.19
Inflammatory Bowel Disease	108	1.89
Heart Disease	90	1.58
Cystitis	87	1.53
Asthma	75	1.32

Behavioural Issues	73	1.28
Obesity	71	1.25
Diabetes	66	1.16
Chronic Diarrhoea	47	0.82
FIV	46	0.81
Urinary Stones	46	0.81
Cancer	44	0.77
Pancreatitis	30	0.53
Chronic Vomiting	27	0.47
Blindness	25	0.44
Liver Disease	18	0.32
Epilepsy	13	0.23
FeLV	2	0.04

Table S7: Variable inclusion frequency across bootstrapped samples

7A. Overweight

Variable	Frequency
Breed	1.00
Sex	1.00
Sex × Neuter Status Interaction	1.00
Daily Dry Food Calories	1.00
Daily Wet Food Calories	1.00
Face Shape	1.00
Free Feeding	1.00
Litter history	1.00
Neuter Status	1.00
Additional Calories from Household Members	1.00

Additional Calories from Neighbours	1.00
Additional Calories from Other Cat Food	1.00
Additional Calories from Stolen Food	1.00
Additional Calories from Table Scraps	1.00
Cats in Household	1.00
Dogs in Household	1.00
Outdoor Space Access	1.00
Pickiness	1.00
Play Frequency	1.00
Probiotic/Prebiotic Supplementation	1.00
Age Group	1.00
Time Spent Outdoors	1.00

Frequency of Food Given Outside Regular Mealtimes	1.00
Cat Tower/Climbing Frame Enrichment	0.83
Sleep Duration	0.63
Number of Other Adults in Household	0.50
Water Intake Frequency	0.50
Birth Environment	0.33
Birth Surroundings	0.27
Stress Frequency	0.27
Additional Calories from Garbage	0.23
Number of Children in Household	0.07

7B. Underweight

Variable	Frequency

Breed	1.00
Sex	1.00
Sex × Neuter Status Interaction	1.00
Daily Fresh Food Calories	1.00
Daily Wet Food Calories	1.00
Scratching Post/Board Enrichment	1.00
Face Shape	1.00
Meals per Day	1.00
Neuter Status	1.00
Additional Calories from Household Members	1.00
Additional Calories from Other Cat Food	1.00
Additional Calories from Table Scraps	1.00

Cats in Household	1.00
Dogs in Household	1.00
Outdoor Space Access	1.00
Pickiness	1.00
Play Frequency	1.00
Sleep Duration	1.00
Probiotic/Prebiotic Supplementation	1.00
Vitamin Supplementation	1.00
Age Group	1.00
Time Spent Outdoors	1.00
Crunchy Treat	1.00
Freeze-Dried Treat	1.00

Lickable Treat	1.00
Pâté Treat	1.00
Prescription/Therapeutic Treat	1.00
Water Intake	1.00
Daily Dry Food Calories	0.97
String/Wand Toy Enrichment	0.97
Additional Calories from Hunted Food	0.97
Additional Calories from Garbage	0.90
Number of Children in Household	0.90
Timed Feeder Use	0.87
Play Duration	0.77
Number of Other Adults in Household	0.67

Cat Tower/Climbing Frame Enrichment	0.60
Additional Calories from Stolen Food	0.50
Home-Cooked Food	0.43
Free Feeding	0.37
Human Food/Table Scrap Treat	0.37
Outdoor Environment Type	0.33
Therapeutic Diet	0.30
Additional Calories from Neighbours	0.13
Window Seat/Perch Enrichment	0.10
Tunnel/Hideout Enrichment	0.07
Daily Raw Food Calories	0.03
Stress Frequency	0.03

7C. Ideal weight model

Variable	Frequency
Breed	1.00
Sex	1.00
Sex × Neuter Status Interaction	1.00
Daily Fresh Food Calories	1.00
Daily Raw Food Calories	1.00
Cat Tower/Climbing Frame Enrichment	1.00
Face Shape	1.00
Free Feeding	1.00
Neuter Status	1.00
Additional Calories from Garbage	1.00
Additional Calories from Hunted Food	1.00

Additional Calories from Neighbours	1.00
Play Frequency	1.00
Stress Frequency	1.00
Vitamin Supplementation	1.00
Age Group	1.00
Frequency of Food Given Outside Regular Mealtimes	1.00
Crunchy Treat	1.00
Freeze-Dried Treat	1.00
Pâté Treat	1.00
Prescription/Therapeutic Treat	1.00
Soft Treat	1.00
Water Intake Frequency	1.00

Time Spent Outdoors	0.97
Daily Dry Food Calories	0.73
Lickable Treat	0.73
Sleep Duration	0.70
Window Seat/Perch Enrichment	0.67
Additional Calories from Other Cat Food	0.67
Birth Surroundings	0.60
Play Duration	0.60
Food Delivery Method	0.47
Indoor Space	0.47
Cats in Household	0.33
Additional Calories from Table Scraps	0.30

Other Dogs in Household	0.30
Home-Cooked Food	0.20
Additional Calories from Stolen Food	0.20
String/Wand Toy Enrichment	0.07
Therapeutic Diet	0.07
Birth Environment	0.03
Pickiness	0.03

Table S8: Model diagnostics

Overweight

Variable	Mean VIF	Max VIF
Sex × Neuter Status Interaction	7.75	7.76
Sex	7.69	7.70
Neuter Status	1.41	1.41
Cats in Household	1.17	1.18
Outdoor Space Access	1.17	1.17
Time Spent Outdoors	1.17	1.17
Additional Calories from Other Cat Food	1.14	1.15
Additional Calories from Neighbours	1.08	1.08
Face Shape	1.06	1.06

Additional Calories from Stolen Food	1.05	1.05
Age Group	1.04	1.04
Free Feeding	1.04	1.04
Litter history	1.04	1.04
Daily Dry Food Calories	1.04	1.04
Additional Calories from Household Members	1.03	1.03
Additional Calories from Table Scraps	1.03	1.03
Play Frequency	1.02	1.03
Pickiness	1.02	1.02
Daily Wet Food Calories	1.02	1.02
Probiotic/Prebiotic Supplementation	1.02	1.02
Breed	1.02	1.02

Other Dogs in Household	1.01	1.01
Food Given Outside Regular Mealtimes	1.01	1.01

Underweight

Variable	Mean VIF	Max VIF
Sex × Neuter Status Interaction	4.31	4.33
Sex	4.20	4.22
Neuter Status	1.49	1.49
Daily Fresh Food Calories	1.33	1.34
Daily Wet Food Calories	1.27	1.28
Scratching Post/Board Enrichment	1.21	1.21
Outdoor Space Access	1.18	1.18
Time Spent Outdoors	1.17	1.17

Cats in Household	1.13	1.14
Additional Calories from Other Cat Food	1.11	1.12
Age Group	1.10	1.11
Meals per Day	1.10	1.10
Face Shape	1.09	1.09
Freeze-Dried Treat	1.09	1.09
Sleep Duration	1.08	1.08
Crunchy Treat	1.08	1.08
Probiotic/Prebiotic Supplementation	1.05	1.05
Play Frequency	1.04	1.05
Vitamin Supplementation	1.04	1.04
Water Intake	1.04	1.04

Lickable Treat	1.04	1.04
Pickiness	1.03	1.03
Breed	1.03	1.03
Other Dogs in Household	1.02	1.02
Additional Calories from Table Scraps	1.02	1.02
Additional Calories from Household Members	1.02	1.02
Pâté Treat	1.01	1.01
Prescription/Therapeutic Treat	1.01	1.01

Ideal weight

Variable	Mean VIF	Max VIF
Sex × Neuter Status Interaction	4.92	4.92
Sex	4.81	4.81

Neuter Status	1.50	1.50
Additional Calories from Hunted Food	1.18	1.18
Cat Tower/Climbing Frame Enrichment	1.09	1.09
Additional Calories from Neighbours	1.09	1.09
Face Shape	1.08	1.08
Age Group	1.07	1.07
Stress Frequency	1.07	1.07
Freeze-Dried Treat	1.06	1.06
Crunchy Treat	1.06	1.06
Daily Fresh Food Calories	1.05	1.06
Additional Calories from Garbage	1.03	1.04
Play Frequency	1.03	1.03

Free Feeding	1.03	1.03
Water Intake Frequency	1.03	1.03
Breed	1.02	1.02
Vitamin Supplementation	1.02	1.02
Soft Treat	1.01	1.01
Daily Raw Food Calories	1.01	1.01
Pâté Treat	1.01	1.01
Food Given Outside Regular Mealtimes	1.01	1.01
Prescription/Therapeutic Treat	1.01	1.01

Cook’s distance summary

Model	Min	1st Quartile	Median	Mean	3rd Quartile	Max
Overweight	0.0063	0.0065	0.0066	0.0066	0.0067	0.0069

Underweight	0.0208	0.0208	0.0209	0.0209	0.0210	0.0213
Ideal Weight	0.0079	0.0080	0.0080	0.0080	0.0081	0.0081

Hosmer-Lemeshow test summary

Model	Min p-value	1st Quartile	Median	Mean	3rd Quartile	Max p-value
Overweight	0.1613	0.6227	0.7267	0.7020	0.7928	0.9532
Underweight	0.0814	0.2040	0.4165	0.4181	0.5752	0.8160
Ideal Weight	0.0242	0.0783	0.1205	0.2205	0.2667	0.7512

Table S9: Robustness checks: owner perception and weight-control behavior

Logistic regression models of owner-perceived weight status outcomes

Variable	Overweight OR (95% CI)	p-value	Underweight OR (95% CI)	p-value	Ideal OR (95% CI)
Food type					
Fresh vs Dry	0.61 (0.12–11.1)	0.63	1.15 (0.23–21.1)	0.89	3.05 (1.26–7.97)
Wet vs Dry	0.52 (0.05–11.4)	0.6	4.16 (0.71–79.5)	0.19	2.30 (0.78–7.16)
BCS-derived status					
Overweight	92.2 (12.3–2040)	<0.001	—	—	—
Underweight	—	—	11.5 (0.79–312)	0.084	—
Ideal	—	—	—	—	16.6 (3.37–128)
Survey age group					
Mature adult vs Kitten	9.22 (1.75–171)	0.036	2.93 (0.72–19.8)	0.18	0.09 (0.02–0.26)
Senior vs Kitten	8.42 (1.65–154)	0.042	23.7 (7.17–147)	<0.001	0.05 (0.01–0.13)
Young adult vs Kitten	7.22 (1.41–133)	0.059	2.63 (0.75–16.7)	0.2	0.14 (0.03–0.38)
Interaction: Food type × BCS					
Fresh × BCS	0.41 (0.02–3.21)	0.47	0.97 (0.04–14.4)	0.98	0.43 (0.05–2.15)
Wet × BCS	0.62 (0.02–10.4)	0.74	0.45 (0.01–8.18)	0.6	0.23 (0.03–1.48)

Type III analysis of deviance across models

Variable	Overweight χ^2 (p)	Underweight χ^2 (p)	Ideal χ^2 (p)		
Food type	0.26 (0.88)	10.82 (0.004)	6.72 (0.035)		
BCS-derived status	26.56 (<0.001)	3.22 (0.073)	13.16 (<0.001)		
Survey age group	8.66 (0.034)	169.63 (<0.001)	120.89 (<0.001)		
Food type × BCS	0.78 (0.68)	1.60 (0.45)	2.69 (0.26)		

Logistic regression model of owner-reported weight control behavior

Variable	Odds Ratio	95% CI	p-value		
Intercept	0.54	0.23–1.30	0.155		
Food type					
Fresh vs Dry	0.52	0.24–1.06	0.083		
Wet vs Dry	0.43	0.18–0.96	0.045		
Body condition status					
Overweight/obese vs ideal weight	3.17	2.50–4.03	<0.001		
Underweight vs ideal weight	0.8	0.62–1.01	0.063		
Survey age group					
Mature adult vs kitten	3.57	2.15–6.08	<0.001		
Senior vs kitten	4.88	3.09–7.97	<0.001		
Young adult vs kitten	2.87	1.82–4.68	<0.001		

Type III analysis of deviance (likelihood ratio tests)					
Variable	χ^2	df	p-value		
Food type	4.23	2	0.12		
Body condition status	121.39	2	<0.001		
Survey age group	63.02	3	<0.001		

Table S10: Alternate overweight and ideal weight models (BCS 6 coded as ideal)

Multivariable logistic regression model examining factors associated with overweight body condition in cats (N = 5,653) with BCS 6 coded as ideal. Odds ratios (ORs), 95% confidence intervals (CIs), and p-values are presented for retained variables in the final overweight versus non-overweight model. Reference categories are indicated as “Ref”. ORs >1 indicate increased odds of being overweight, whereas ORs <1 indicate reduced odds of being overweight relative to the reference category. P-values and 95% CIs are nominal and unadjusted for multiple comparisons; because predictors were retained through data-driven selection, estimates are conditional on the selected model and should be interpreted as exploratory.

Variable		OR [CI]	p
Age group	Young adult	Ref	
	Kitten	0.32 [0.17–0.61]	<0.001
	Mature adult	1.93 [1.54–2.41]	<0.001
	Senior	1.48 [1.20–1.83]	<0.001
	Geriatric	0.71 [0.52–0.95]	0.022
Birth surroundings	Suburban	Ref	
	Rural	0.88 [0.68–1.15]	>0.05
	Unknown	0.91 [0.74–1.12]	>0.05
	Urban	0.81 [0.65–1.02]	>0.05
Breed	Domestic Shorthair	Ref	
	Asian group	0.74 [0.37–1.49]	>0.05
	Bengal	0.71 [0.38–1.33]	>0.05
	British Shorthair	1.67 [1.25–2.23]	<0.001
	Domestic Long Hair	1.36 [0.96–1.92]	>0.05
	Domestic Medium Hair	1.41 [1.05–1.91]	0.024
	Hybrid group	9.04 [2.59–31.58]	<0.001
	Maine Coon	0.68 [0.34–1.34]	>0.05
	Mediterranean group	6.00 [1.60–22.53]	0.008
	Mixed	1.31 [1.06–1.63]	0.014
	Other purebred	1.79 [1.30–2.48]	<0.001
	Ragdoll	0.46 [0.25–0.84]	0.011
	Western group	1.10 [0.73–1.67]	>0.05
Sex	Female	Ref	
	Male	0.94 [0.22–4.06]	>0.05
Neutering	No	Ref	
	Yes	2.62 [0.91–7.54]	>0.05
Sex × Neutered	Interaction	1.24 [0.29–5.34]	>0.05
Feeding	Owner-estimated proportion of meal calories from dry food	1.87 [1.27–2.75]	0.002
	Owner-estimated proportion of meal calories from wet food	0.67 [0.47–0.96]	0.029
	Free feeding	0.63 [0.47–0.83]	0.001
	Fed additional calories from other household members	1.36 [1.04–1.78]	0.024
	Fed additional calories from neighbours	1.55 [1.16–2.06]	0.003
	Eats other cats' food	1.55 [1.23–1.95]	<0.001
	Gets additional calories from unknown sources	1.42 [1.02–1.98]	0.036
	Fed freeze-dried treats	0.84 [0.82–0.90]	0.002
Face shape	Mesocephalic	Ref	
	Brachycephalic	1.21 [0.69–2.11]	>0.05
	Dolichocephalic	0.33 [0.23–0.46]	<0.001

Litter number	None (or male)	Ref	
	One	1.67 [1.14–2.44]	0.009
	Two or more	1.46 [0.86–2.49]	>0.05
	Unsure	1.02 [0.71–1.45]	>0.05
Meals per day	Not meal fed	Ref	
	One or two	1.02 [0.77–1.34]	>0.05
	Three or more	1.16 [0.85–1.57]	>0.05
Maximum outdoor space access	None	Ref	
	Porch	0.99 [0.69–1.43]	>0.05
	Yard	1.16 [0.83–1.63]	>0.05
	Free-roaming	0.99 [0.68–1.45]	>0.05
	Other	0.60 [0.37–0.96]	0.035
Cat's pickiness about food	Not picky	Ref	
	Slightly picky	0.73 [0.60–0.89]	0.001
	Very picky	0.70 [0.55–0.88]	0.002
Play frequency	Once a day or less	Ref	
	Twice a day	0.81 [0.64–1.02]	>0.05
	Three times a day	0.91 [0.69–1.22]	>0.05
	Four times a day or more	0.83 [0.65–1.07]	>0.05
Daily sleep	<10 hours	Ref	
	10–16 hours	1.16 [0.91–1.48]	>0.05
	16+ hours	1.49 [1.14–1.95]	0.004
Time outdoors	None	Ref	
	1–25%	1.01 [0.76–1.35]	>0.05
	25–50%	0.93 [0.67–1.29]	>0.05
	50–75%	0.89 [0.61–1.31]	>0.05
	75% or more	0.40 [0.22–0.75]	0.004
Household	Number of dogs in household	0.78 [0.65–0.94]	0.008
	Cat tower/climbing frame access	0.80 [0.67–0.96]	0.015
	Access to outdoors unsupervised	0.79 [0.59–1.06]	>0.05

Multivariable logistic regression model examining factors associated with ideal body condition in cats (N = 5,653) with BCS 6 coded as ideal. Odds ratios (ORs), 95% confidence intervals (CIs), and p-values are presented for retained variables in the final ideal weight versus non-ideal model. Reference categories are indicated as “Ref”. ORs >1 indicate increased odds of being ideal weight, whereas ORs <1 indicate reduced odds of being ideal weight relative to the reference category. P-values and 95% CIs are nominal and unadjusted for multiple comparisons; because predictors were retained through data-driven selection, estimates are conditional on the selected model and should be interpreted as exploratory.

Variable		OR [CI]	p
Age group	Young adult	Ref	
	Kitten	0.89 [0.68–1.17]	>0.05
	Mature adult	0.68 [0.57–0.81]	<0.001
	Senior	0.68 [0.58–0.79]	<0.001
	Geriatric	0.58 [0.48–0.71]	<0.001
Breed	Domestic Shorthair	Ref	
	Asian group	0.71 [0.51–0.99]	0.043
	Bengal	0.76 [0.55–1.05]	>0.05
	British Shorthair	0.91 [0.72–1.16]	>0.05
	Domestic Long Hair	0.97 [0.74–1.27]	>0.05
	Domestic Medium Hair	1.09 [0.86–1.38]	>0.05
	Hybrid group	0.39 [0.13–1.19]	>0.05
	Maine Coon	1.28 [0.86–1.89]	>0.05
	Mediterranean group	0.44 [0.12–1.55]	>0.05
	Mixed	0.98 [0.84–1.15]	>0.05
	Other purebred	0.76 [0.59–0.97]	0.028
	Ragdoll	1.31 [0.94–1.81]	>0.05
	Western group	1.06 [0.79–1.41]	>0.05

Sex	Female	Ref	
	Male	2.08 [1.23–3.53]	0.006
Neutering	Not neutered	Ref	
	Neutered	1.62 [1.09–2.42]	0.017
Sex × Neutered	Interaction	0.49 [0.29–0.84]	0.010
Feeding	Owner-estimated proportion of meal calories from fresh food	1.24 [1.05–1.47]	0.011
	Owner-estimated proportion of meal calories from raw food	2.00 [0.85–4.71]	>0.05
	Free feeding	1.12 [0.96–1.30]	>0.05
	Eats from garbage	1.93 [1.09–3.42]	0.025
	Hunts food	1.10 [0.94–1.28]	>0.05
	Fed additional calories from neighbours	0.78 [0.62–0.98]	0.031
	Given vitamins	0.74 [0.56–0.98]	0.037
	Fed crunchy treats	1.16 [1.03–1.30]	0.018
	Fed freeze-dried treats	1.33 [1.16–1.52]	<0.001
	Fed pate treats	0.55 [0.33–0.91]	0.020
Face shape	Mesocephalic	Ref	
	Brachycephalic	0.66 [0.43–1.02]	>0.05
	Dolichocephalic	0.90 [0.75–1.07]	>0.05
Cat's pickiness about food	Not picky	Ref	
	Slightly picky	0.94 [0.81–1.08]	>0.05
	Very picky	0.88 [0.75–1.05]	>0.05

Play frequency	Once a day or less	Ref	
	Twice a day	1.16 [0.99–1.37]	>0.05
	Three times a day	0.98 [0.80–1.20]	>0.05
	Four times a day or more	1.02 [0.85–1.21]	>0.05
Observed water frequency	Less than daily	Ref	
	Daily	1.06 [0.92–1.23]	>0.05
	More than daily	0.87 [0.75–1.01]	>0.05
Household	Number of cats in household	0.89 [0.83–0.96]	0.004
	Scratching post/board access	1.12 [0.96–1.30]	>0.05
	Window seat/perch access	1.15 [1.00–1.32]	0.044
	Therapeutic diet	0.84 [0.69–1.03]	>0.05

Table S11: Alternate overweight, underweight, and ideal weight models (kittens excluded)

Multivariable logistic regression model examining factors associated with overweight body condition in adult cats (N = 5,294) with kittens less than one year old removed (N = 359). Odds ratios (ORs), 95% confidence intervals (CIs), and p-values are presented for retained variables in the final overweight versus non-overweight model. Reference categories are indicated as “Ref”. ORs >1 indicate increased odds of being overweight, whereas ORs <1 indicate reduced odds of being overweight relative to the reference category. P-values and 95% CIs are nominal and unadjusted for multiple comparisons; because predictors were retained through data-driven selection, estimates are conditional on the selected model and should be interpreted as exploratory.

Variable		OR [95% CI]	p
Age group	Young adult	Ref	—
	Mature adult	1.72 [1.43–2.08]	<0.001
	Senior	1.38 [1.17–1.62]	<0.001
	Geriatric	0.72 [0.58–0.89]	0.003
Birth surroundings	Suburban	Ref	—
	Rural	0.93 [0.75–1.15]	>0.05
	Unknown	1.04 [0.87–1.23]	>0.05
	Urban	0.85 [0.70–1.02]	>0.05
Breed	Domestic Shorthair	Ref	—
	Asian	0.79 [0.49–1.28]	>0.05
	Bengal	0.57 [0.35–0.93]	0.023
	British Shorthair	1.76 [1.38–2.26]	<0.001
	Domestic Longhair	1.27 [0.95–1.70]	>0.05
	Domestic Medium Hair	1.39 [1.08–1.79]	0.011
	Hybrid	3.18 [0.94–10.81]	>0.05
	Maine Coon	0.62 [0.37–1.03]	>0.05

	Mediterranean	3.50 [0.91–13.52]	>0.05
	Mixed	1.26 [1.06–1.50]	0.01
	Other	1.26 [0.95–1.67]	>0.05
	Ragdoll	0.38 [0.24–0.60]	<0.001
	Western	0.98 [0.71–1.36]	>0.05
Sex	Female	Ref	—
	Male	0.44 [0.14–1.41]	>0.05
Neutered	No	Ref	—
	Yes	1.40 [0.69–2.85]	>0.05
Sex × neutered	Interaction	2.93 [0.91–9.47]	>0.05
Feeding	Owner-estimated proportion of meal calories from dry food	1.81 [1.31–2.49]	<0.001
	Owner-estimated proportion of meal calories from wet food	0.69 [0.52–0.92]	0.01
	Free feeding	0.62 [0.51–0.75]	<0.001
	Fed additional calories from other household members	1.24 [0.99–1.56]	>0.05
	Fed additional calories from neighbours	1.43 [1.11–1.83]	0.005
	Eats other cats' food	1.53 [1.23–1.90]	<0.001
	Steals food	1.24 [0.97–1.59]	>0.05
	Gets table scraps	1.31 [1.06–1.61]	0.013
	Fed additional cat food outside mealtimes as treats	1.46 [1.02–2.08]	0.036
Pickiness	Not picky	Ref	—
	Slightly picky	0.85 [0.72–1.00]	0.049
	Very picky	0.71 [0.59–0.86]	<0.001

Supplements	Probiotic or prebiotic supplements	0.79 [0.61–1.04]	>0.05
Face shape	Mesocephalic	Ref	—
	Brachycephalic	1.06 [0.66–1.71]	>0.05
	Dolichocephalic	0.41 [0.32–0.53]	<0.001
Litter history	None	Ref	—
	One	1.44 [1.03–2.00]	0.03
	Two or more	1.39 [0.88–2.20]	>0.05
	Unsure	1.03 [0.77–1.38]	>0.05
Maximum outdoor space access	None	Ref	—
	Free roaming	0.98 [0.76–1.26]	>0.05
	Other	1.00 [0.72–1.40]	>0.05
	Porch	1.38 [1.03–1.83]	0.028
	Yard	1.36 [1.05–1.76]	0.019
Play frequency	Once a day or less	Ref	—
	Twice a day	0.78 [0.65–0.94]	0.008
	Three times a day	0.73 [0.57–0.93]	0.01
	Four times or more a day	0.80 [0.65–0.97]	0.026
Time outdoors	None	Ref	—
	1–25%	0.90 [0.71–1.13]	>0.05
	25–50%	0.81 [0.62–1.05]	>0.05
	50–75%	0.62 [0.46–0.84]	0.002
	≥75%	0.63 [0.41–0.96]	0.031

Household	Number of cats	0.88 [0.79–0.97]	0.01
	Number of dogs	0.82 [0.71–0.95]	0.007

Multivariable logistic regression model examining factors associated with underweight body condition in adult cats (N = 5,294) with kittens less than one year old removed (N = 359). Odds ratios (ORs), 95% confidence intervals (CIs), and p-values are presented for retained variables in the final underweight versus non-underweight model. Reference categories are indicated as “Ref”. ORs >1 indicate increased odds of being underweight, whereas ORs <1 indicate reduced odds of being underweight relative to the reference category.P-values and 95% CIs are nominal and unadjusted for multiple comparisons; because predictors were retained through data-driven selection, estimates are conditional on the selected model and should be interpreted as exploratory.

Variable		OR [95% CI]	p
Age group	Young adult	Ref	—
	Mature adult	0.86 [0.67–1.09]	>0.05
	Senior	1.10 [0.91–1.34]	>0.05
	Geriatric	2.20 [1.75–2.76]	<0.001
Breed	Domestic Shorthair	Ref	—
	Asian	1.26 [0.86–1.86]	>0.05
	Bengal	1.59 [1.09–2.31]	0.015
	British Shorthair	0.58 [0.41–0.83]	0.003
	Domestic Longhair	0.79 [0.56–1.11]	>0.05
	Domestic Medium Hair	0.64 [0.46–0.89]	0.008
	Hybrid	0.46 [0.10–2.19]	>0.05
	Maine Coon	0.77 [0.45–1.31]	>0.05
	Mediterranean	0.58 [0.07–4.79]	>0.05
	Moggie	0.83 [0.67–1.02]	>0.05

	Other	0.96 [0.70–1.32]	>0.05
	Ragdoll	1.11 [0.74–1.66]	>0.05
	Western	0.79 [0.54–1.16]	>0.05
Sex	Female	Ref	—
	Male	0.52 [0.22–1.24]	>0.05
Neutered	No	Ref	—
	Yes	0.46 [0.25–0.86]	0.015
Sex × neutered	Male × Yes	1.61 [0.67–3.86]	>0.05
Feeding	Owner-estimated proportion of meal calories from dry food	0.57 [0.37–0.88]	0.012
	Owner-estimated proportion of meal calories from fresh food	0.71 [0.52–0.95]	0.023
	Owner-estimated proportion of meal calories from wet food	1.14 [0.78–1.68]	>0.05
	Fed additional calories from other household members	0.72 [0.52–0.98]	0.039
	Steals other cats' food	0.70 [0.53–0.92]	0.009
	Gets table scraps	0.76 [0.58–1.00]	>0.05
	Receives crunchy treats	0.85 [0.72–0.99]	0.04
	Receives freeze-dried treats	0.78 [0.65–0.93]	0.007
	Receives human food or table scraps as treats	1.21 [0.94–1.55]	>0.05
	Receives lickable treats	0.83 [0.68–1.01]	>0.05
	Receives pâté treats	1.91 [1.04–3.52]	0.038
Food delivery method	Traditional	Ref	—
	Electronic	0.95 [0.63–1.44]	>0.05
	Hand fed	0.80 [0.29–2.19]	>0.05

	Other	0.81 [0.62–1.07]	>0.05
	Toy	0.45 [0.17–1.13]	>0.05
Pickiness	Not picky	Ref	—
	Slightly picky	1.54 [1.26–1.89]	<0.001
	Very picky	1.71 [1.37–2.14]	<0.001
Supplements	Probiotics or prebiotics	1.30 [0.98–1.73]	>0.05
	Vitamins	1.54 [1.09–2.17]	0.014
Observed water intake frequency	Less than once daily	Ref	—
	Daily	1.10 [0.90–1.34]	>0.05
	More than daily	1.33 [1.08–1.63]	0.006
Face shape	Mesocephalic	Ref	—
	Brachycephalic	1.91 [1.11–3.28]	0.019
	Dolichocephalic	2.20 [1.79–2.72]	<0.001
Time outdoors	None	Ref	—
	1–25%	0.89 [0.72–1.09]	>0.05
	25–50%	1.11 [0.89–1.39]	>0.05
	50–75%	1.16 [0.89–1.51]	>0.05
	≥75%	1.83 [1.24–2.69]	0.002
Household	Scratching post or board	0.78 [0.64–0.95]	0.015
	Number of cats	1.21 [1.10–1.34]	<0.001
	Number of dogs	1.25 [1.10–1.43]	<0.001

Multivariable logistic regression model examining factors associated with ideal body condition in adult cats (N = 5,294) with kittens less than one year old removed (N = 359). Odds ratios (ORs), 95% confidence intervals (CIs), and p-values are presented for retained variables in the final ideal versus non-ideal model. Reference categories are indicated as “Ref”. ORs >1 indicate increased odds of being at an ideal body condition, whereas ORs <1 indicate reduced odds of being ideal relative to the reference category.

Variable		OR [95% CI]	p
Age group	Young adult	Ref	—
	Mature adult	0.68 [0.57–0.81]	<0.001
	Senior	0.68 [0.59–0.80]	<0.001
	Geriatric	0.59 [0.48–0.71]	<0.001
Birth surroundings	Suburban	Ref	—
	Rural	1.06 [0.88–1.28]	>0.05
	Unknown	1.05 [0.90–1.22]	>0.05
	Urban	1.10 [0.93–1.30]	>0.05
Breed	Domestic Shorthair	Ref	—
	Asian	0.80 [0.56–1.13]	>0.05
	Bengal	0.70 [0.50–0.99]	0.043
	British Shorthair	0.98 [0.77–1.25]	>0.05
	Domestic Longhair	0.97 [0.74–1.26]	>0.05
	Domestic Medium Hair	1.07 [0.84–1.37]	>0.05
	Hybrid	0.39 [0.13–1.19]	>0.05
	Maine Coon	1.29 [0.84–1.99]	>0.05
	Mediterranean	0.58 [0.15–2.23]	>0.05
	Moggie	0.98 [0.83–1.15]	>0.05

	Other	0.74 [0.57–0.96]	0.021
	Ragdoll	1.29 [0.91–1.84]	>0.05
	Western	1.14 [0.84–1.54]	>0.05
Sex	Female	Ref	—
	Male	1.83 [0.85–3.91]	>0.05
Neutered	No	Ref	—
	Yes	1.50 [0.87–2.61]	>0.05
Sex × neutered	Male × Yes	0.56 [0.26–1.21]	>0.05
Feeding	Owner-estimated proportion of meal calories from fresh food	1.27 [1.07–1.50]	0.006
	Free feeding	1.11 [0.95–1.30]	>0.05
	Eats from garbage	1.93 [1.07–3.49]	0.029
	Hunts food	1.13 [0.95–1.34]	>0.05
	Receives additional calories from neighbors	0.79 [0.62–0.99]	0.045
	Steals other cats' food	0.84 [0.70–1.01]	>0.05
	Fed additional cat food outside mealtimes as treats	0.98 [0.70–1.36]	>0.05
	Receives crunchy treats	1.21 [1.07–1.37]	0.002
	Receives freeze-dried treats	1.42 [1.23–1.63]	<0.001
	Receives pâté treats	0.63 [0.37–1.06]	>0.05
	Receives prescription/therapeutic treats	1.44 [0.78–2.65]	>0.05
	Receives soft treats	1.12 [0.94–1.33]	>0.05
Supplements	Vitamins	0.74 [0.55–0.99]	0.042
Observed water intake frequency	Less than once daily	Ref	—

	Daily	1.03 [0.89–1.20]	>0.05
	More than daily	0.84 [0.72–0.98]	0.027
Face shape	Mesocephalic	Ref	—
	Brachycephalic	0.64 [0.41–1.00]	0.049
	Dolichocephalic	0.87 [0.73–1.05]	>0.05
Household	Cat tower or climbing frame	1.12 [0.99–1.27]	>0.05
Play frequency	Once a day or less	Ref	—
	Twice a day	1.19 [1.01–1.41]	0.041
	Three times a day	0.99 [0.80–1.23]	>0.05
	Four times or more a day	1.06 [0.88–1.27]	>0.05
Time outdoors	None	Ref	—
	1–25%	1.08 [0.93–1.26]	>0.05
	25–50%	1.00 [0.83–1.19]	>0.05
	50–75%	1.03 [0.82–1.29]	>0.05
	≥75%	0.92 [0.65–1.29]	>0.05

Table S12: Multinomial regression results

Multinomial logistic regression results examining factors associated with underweight and overweight body condition in cats relative to ideal weight (N = 5,653). Reference category for all odds ratios is ideal body condition.

Underweight vs Ideal

Variable		OR [CI]	p
Age group	Young adult	Ref	
	Kitten	1.45 [1.06–1.96]	0.018
	Mature adult	1.09 [0.85–1.41]	>0.05
	Senior	1.33 [1.08–1.63]	0.007
	Geriatric	2.28 [1.78–2.91]	<0.001
Breed	Domestic Shorthair	Ref	
	Asian group	1.41 [0.96–2.06]	>0.05
	Bengal	1.31 [0.90–1.89]	>0.05
	British Shorthair	0.81 [0.58–1.15]	>0.05
	Domestic Long Hair	0.84 [0.59–1.20]	>0.05
	Domestic Medium Hair	0.72 [0.51–1.00]	0.051
	Hybrid group	0.67 [0.13–3.49]	>0.05
	Maine Coon	0.72 [0.44–1.17]	>0.05
	Mediterranean group	1.04 [0.10–10.98]	>0.05
	Mixed	0.87 [0.70–1.07]	>0.05
	Other purebred	1.02 [0.74–1.41]	>0.05
	Ragdoll	0.80 [0.54–1.18]	>0.05
	Western group	0.88 [0.61–1.27]	>0.05
Sex	Female	Ref	
	Male	0.42 [0.23–0.76]	0.004
Neutering	Not neutered	Ref	
	Neutered	0.52 [0.33–0.81]	0.004
Sex × Neutered	Interaction	2.15 [1.16–3.95]	0.014
Feeding	Owner-estimated proportion of meal calories from fresh food	0.71 [0.52–0.96]	0.026
	Owner-estimated proportion of meal calories from raw food	0.44 [0.16–1.24]	>0.05
	Owner-estimated proportion of meal calories from wet food	1.11 [0.76–1.63]	>0.05
	Owner-estimated proportion of meal calories from dry food	0.75 [0.48–1.17]	>0.05
	Fed additional calories from neighbours	1.05 [0.76–1.44]	>0.05
	Eats other cats' food	0.81 [0.62–1.06]	>0.05
	Steals food	0.87 [0.62–1.22]	>0.05
	Gets table scraps	0.86 [0.65–1.13]	>0.05
	Fed additional calories from other household members	0.76 [0.55–1.04]	>0.05
	Fed crunchy treats	0.84 [0.71–0.98]	0.030
	Fed freeze-dried treats	0.80 [0.67–0.96]	0.016
	Fed pate treats	2.50 [1.34–4.66]	0.004

	Fed additional cat food outside mealtimes as treats	1.20 [0.78–1.85]	>0.05
Face shape	Mesocephalic	Ref	
	Brachycephalic	1.91 [1.09–3.34]	0.025
	Dolichocephalic	1.72 [1.40–2.11]	<0.001
Food delivery method	Traditional	Ref	
	Electronic	0.91 [0.60–1.39]	>0.05
	Hand-fed	1.05 [0.39–2.84]	>0.05
	Other	0.67 [0.49–0.92]	0.013
	Toy	0.50 [0.22–1.13]	>0.05
Meals per day	Not meal fed	Ref	
	One or two	0.76 [0.57–1.01]	>0.05
	Three or more	0.67 [0.49–0.92]	0.013
Maximum outdoor space access	None	Ref	
	Porch	0.83 [0.59–1.18]	>0.05
	Yard	0.91 [0.70–1.20]	>0.05
	Free-roaming	0.96 [0.72–1.29]	>0.05
	Other	1.12 [0.78–1.59]	>0.05
Cat's pickiness about food	Not picky	Ref	
	Slightly picky	1.47 [1.20–1.79]	<0.001
	Very picky	1.57 [1.25–1.96]	<0.001
Play frequency	Once a day or less	Ref	
	Twice a day	0.88 [0.71–1.09]	>0.05
	Three times a day	1.07 [0.82–1.39]	>0.05
	Four times a day or more	1.15 [0.92–1.43]	>0.05
Supplements	Probiotics or prebiotics	1.25 [0.94–1.65]	>0.05
	Vitamins	1.57 [1.10–2.24]	0.013
Time outdoors	None	Ref	
	1–25%	0.90 [0.69–1.18]	>0.05
	25–50%	1.08 [0.80–1.46]	>0.05
	50–75%	1.01 [0.72–1.42]	>0.05
	75% or more	1.69 [1.10–2.60]	0.017
Observed water frequency	Less than daily	Ref	
	Daily	1.01 [0.83–1.23]	>0.05
	More than daily	1.25 [1.02–1.53]	0.029
Household	Number of cats in household	1.18 [1.06–1.31]	0.002
	Number of dogs in household	1.24 [1.09–1.42]	0.001
	Cat tower/climbing frame access	0.91 [0.77–1.09]	>0.05

Overweight vs. Ideal

Variable		OR [CI]	p
Age group	Young adult	Ref	
	Kitten	0.23 [0.14–0.38]	<0.001
	Mature adult	1.76 [1.45–2.14]	<0.001
	Senior	1.45 [1.22–1.73]	<0.001
	Geriatric	0.90 [0.70–1.15]	>0.05
Breed	Domestic Shorthair	Ref	
	Asian group	0.90 [0.55–1.49]	>0.05
	Bengal	0.62 [0.38–1.01]	>0.05
	British Shorthair	1.64 [1.27–2.12]	<0.001
	Domestic Long Hair	1.21 [0.90–1.63]	>0.05
	Domestic Medium Hair	1.25 [0.96–1.62]	>0.05
	Hybrid group	2.88 [0.81–10.22]	>0.05
	Maine Coon	0.64 [0.39–1.06]	>0.05
	Mediterranean group	4.92 [1.17–20.72]	0.030
	Mixed	1.20 [1.00–1.44]	0.046
	Other purebred	1.28 [0.95–1.71]	>0.05
	Ragdoll	0.44 [0.28–0.68]	<0.001
	Western group	0.93 [0.67–1.30]	>0.05
Sex	Female	Ref	

	Male	0.62 [0.23–1.67]	>0.05
Neutering	Not neutered	Ref	
	Neutered	1.52 [0.75–3.06]	>0.05
Sex × Neutered	Interaction	1.90 [0.70–5.15]	>0.05
Feeding	Owner-estimated proportion of meal calories from dry food	1.72 [1.16–2.53]	0.007
	Owner-estimated proportion of meal calories from fresh food	0.85 [0.63–1.13]	>0.05
	Owner-estimated proportion of meal calories from raw food	0.45 [0.15–1.36]	>0.05
	Owner-estimated proportion of meal calories from wet food	0.62 [0.43–0.90]	0.012
	Free feeding	0.61 [0.47–0.79]	<0.001
	Fed additional calories from neighbours	1.45 [1.12–1.88]	0.004
	Eats other cats' food	1.40 [1.12–1.76]	0.003
	Steals food	1.24 [0.96–1.60]	>0.05
	Gets table scraps	1.28 [1.03–1.59]	0.027
	Fed additional calories from other household members	1.17 [0.92–1.47]	>0.05
	Fed crunchy treats	0.94 [0.82–1.08]	>0.05
	Fed freeze-dried treats	0.87 [0.74–1.01]	>0.05
	Fed pate treats	1.66 [0.87–3.15]	>0.05
	Fed additional cat food outside mealtimes as treats	1.52 [1.05–2.22]	0.028

Face shape	Mesocephalic	Ref	
	Brachycephalic	1.31 [0.79–2.17]	>0.05
	Dolichocephalic	0.49 [0.38–0.63]	<0.001
Food delivery method	Traditional	Ref	
	Electronic	0.76 [0.52–1.11]	>0.05
	Hand-fed	1.27 [0.52–3.07]	>0.05
	Other	0.81 [0.61–1.07]	>0.05
	Toy	0.87 [0.51–1.48]	>0.05
Meals per day	Not meal fed	Ref	
	One or two	0.96 [0.72–1.28]	>0.05
	Three or more	1.03 [0.76–1.39]	>0.05
Maximum outdoor space access	None	Ref	
	Porch	1.33 [0.98–1.80]	>0.05
	Yard	1.34 [1.02–1.75]	0.033
	Free-roaming	0.97 [0.75–1.28]	>0.05
	Other	1.02 [0.72–1.44]	>0.05
Cat's pickiness about food	Not picky	Ref	
	Slightly picky	0.92 [0.78–1.09]	>0.05
	Very picky	0.79 [0.65–0.97]	0.024
Play frequency	Once a day or less	Ref	
	Twice a day	0.75 [0.62–0.91]	0.003

	Three times a day	0.72 [0.57–0.92]	0.011
	Four times a day or more	0.83 [0.67–1.03]	>0.05
Supplements	Probiotics or prebiotics	0.84 [0.63–1.11]	>0.05
	Vitamins	1.15 [0.80–1.65]	>0.05
Time outdoors	None	Ref	
	1–25%	0.85 [0.67–1.09]	>0.05
	25–50%	0.78 [0.60–1.02]	>0.05
	50–75%	0.59 [0.43–0.81]	0.001
	75% or more	0.66 [0.44–1.00]	>0.05
Observed water frequency	Less than daily	Ref	
	Daily	0.86 [0.73–1.02]	>0.05
	More than daily	0.95 [0.79–1.15]	>0.05
Household	Number of cats in household	0.95 [0.86–1.05]	>0.05
	Number of dogs in household	0.87 [0.75–1.01]	>0.05
	Cat tower/climbing frame access	0.87 [0.75–1.01]	>0.05
